# Overexpression of the Transcription Factor Sp1 Activates the OAS-RNAse L-RIG-I Pathway

**DOI:** 10.1371/journal.pone.0118551

**Published:** 2015-03-04

**Authors:** Valéryane Dupuis-Maurin, Lilia Brinza, Joël Baguet, Emilie Plantamura, Stéphane Schicklin, Solène Chambion, Claire Macari, Martine Tomkowiak, Emmanuelle Deniaud, Yann Leverrier, Jacqueline Marvel, Marie-Cécile Michallet

**Affiliations:** Centre International de Recherche en Infectiologie, INSERM U111-CNRS UMR5308, Université de Lyon 1, ENS de Lyon, Lyon, France; University of California at Davis, UNITED STATES

## Abstract

Deregulated expression of oncogenes or transcription factors such as specificity protein 1 (Sp1) is observed in many human cancers and plays a role in tumor maintenance. Paradoxically in untransformed cells, Sp1 overexpression induces late apoptosis but the early intrinsic response is poorly characterized. In the present work, we studied increased Sp1 level consequences in untransformed cells and showed that it turns on an early innate immune transcriptome. Sp1 overexpression does not activate known cellular stress pathways such as DNA damage response or endoplasmic reticulum stress, but induces the activation of the OAS-RNase L pathway and the generation of small self-RNAs, leading to the upregulation of genes of the antiviral RIG-I pathway at the transcriptional and translational levels. Finally, Sp1-induced intrinsic innate immune response leads to the production of the chemokine CXCL4 and to the recruitment of inflammatory cells *in vitro* and *in vivo*. Altogether our results showed that increased Sp1 level in untransformed cells constitutes a novel danger signal sensed by the OAS-RNase L axis leading to the activation of the RIG-I pathway. These results suggested that the OAS-RNase L-RIG-I pathway may be activated in sterile condition in absence of pathogen.

## Introduction

Specificity protein 1 (Sp1) transcription factor, the founding member of the Sp/XKLF (Krüppel-like factor) transcription factor family, is ubiquitously expressed and contains a highly conserved DNA binding domain [1, 2] present in the promoter of many housekeeping genes. Sp1 was thereby first thought to be a constitutive regulator of basal promoters activity. It is now clear that Sp1 regulates a plethora of mammalian genes [3] coding for proteins and also for noncoding RNAs [4], requiring the tight regulation of its expression/activity by multiple post-translational modifications such as phosphorylation, acetylation, glycosylation, ubiquitination and sumoylation [5]. Genetic disruption of *Sp1* leads to embryonic lethality in mice, demonstrating its central role during developmental processes [6]. After birth, Sp1 regulates multiple cellular processes, such as cell cycle progression through for instance transcriptional regulation of p21*waf1/cip1* [7], apoptosis [8–10] or DNA damage response through transcriptional regulation of DDB1 and DDB2 [11]. Sp1 could contribute to tumorigenesis by controlling gene transcription related to cellular growth and proliferation [12]. Sp1 expression is associated with bad prognosis in gastric cancers [13], and has been reported to be overexpressed and/or over-activated in a number of human cancers at late stage. In recent years, it became obvious that Sp1 is also intimately involved in early stages of cellular transformation. We and others have demonstrated that increasing Sp1 concentration in untransformed cells induces a cell cycle G1/S slowdown [14] that is followed by p53-dependent apoptosis [8, 9], clearly recapitulating early protective cellular events occuring upon oncogenic stress. It was also shown that Sp1 level increases during cellular transformation in a fibrosarcoma transformation model [15]. Finally, a recent study demonstrated that Sp1 level accumulated strongly in early stage of lung tumor formation, and that Sp1 expression is maintained at intermediate levels when tumor cells become invasive or malignant [16].

The innate immune response allows the detection of a diverse collection of microbial pathogens such as bacteria, viruses or fungi, based on the recognition of pathogen associated molecular patterns (PAMP) by pattern recognition receptors (PRR). In the advent of a viral infection, immune cells use specific sensors belonging to the PRR family [17] that detect the presence of the virus. The viral detection triggers the initiation of signalling cascades leading to the production of inflammatory cytokines and the synthesis of type I interferons (IFN), which exhibit potent antiviral and immunomodulatory functions [18]. The first identified family of PRR involved in the detection of viral nucleic acids was the Toll-like receptor (TLR) family, including TLR3 located on the cell surface and TRL7, 8 and 9 all confined in endosomal compartments [19]. A second class of PRR, named RIG-like receptors (RLR), has been identified more recently. RLR are cytoplasmic sensors specialized in the detection of viral products that have gained access to the cytosol and three members are known so far: retinoic-acid-inducible gene I (RIG-I), melanoma differentiation-associated gene 5 (MDA5) and laboratory of genetics and physiology-2 (LGP2) [20]. Only RIG-I and MDA5 contain a tandem caspase activation and recruitment domains (CARDs) allowing them to interact with their common adaptor MAVS (also known as CARDIF, IPS-1 and VISA) [21]. The differences between these two helicases RIG-I and MDA5 rely on ligand lenghts preferences [22] and their ability to distinguish different RNA viruses [23]. In the context of RLR signaling, MAVS transmits the signal leading to the activation of Tank binding kinase-1 (TBK-1) and Inhibitor of κB kinase e (IKKe) that can phosphorylate interferon regulating factors (IRF) 3 and 7, as well as nuclear factor kB (NFkB) [24]. Activated, nuclear IRFs and NFkB transcription factors trigger the expression of inflammatory cytokines, type I IFNs and IFN stimulated genes (ISG) that limit viral replication and often lead to viral clearance.

In the present work, we aimed at deciphering the role of Sp1 overexpression in initial steps of cellular transformation. To answer this question, we used cellular models allowing us to induce Sp1 level accumulation in untransformed cells. We demonstrated that Sp1 overepression leads to an early endogenous response that induces the expression of genes associated with an inflammatory network. The cellular response induced by Sp1 overexpression is different from known cellular stress pathways such as DNA damage or endoplasmic reticulum (ER) stress. This intrinsic response is mainly characterized by the induction of the antiviral RIG-I signaling pathway as well as ISGs. We thus characterized the RIG-I dependent response induced by increased Sp1 level, and found that various genes, such as RIG-I, MAVS and IRF3 are upregulated at the transcriptional and translational levels. This early intrinsic response also includes the activation of the OAS-RNase L axis and the generation of small self-RNAs that lead to the activation of the antiviral IRF3 promoter. Finally, Sp1 overexpression is associated to a specific secretome, including CXCL4, allowing the recruitment of inflammatory cells *in vitro* and *in vivo*.

## Materials and Methods

### Microarray functional analysis

Microarray generation and analysis of gene expression (FC> = 1.3) upon Sp1 overexpression was previously described [14]. A more specific Sp1 signature (FC> = 1.5, 1187 unique genes) was defined using “affy” and “limma” Bioconductor/R packages: RMA method [25] was used for background correction, quantile normalization and summarization, while the limma [26] method was used for the differential expression analysis. A False Discovery Rate (FDR—[27]) multiple testing procedure was used to adjust raw p-values and a threshold of 0.05 was used on the adjusted p-values in order to select the differentially expressed genes. A functional analysis of the Sp1 signature was performed with the g:Profiler [28, 29]. g:GOSt (the gene group functional profiling core of the g:Profiler) presents two advantages for studying long gene lists: 1- the use of a custom multiple testing correction procedure (g:SCS) considering the unevenly distributed structure of the functional annotations; 2- the possibility to analyze ordered gene lists which allows to identify both the specific terms associated with the most strong changes and the terms that are characterizing the gene set more generally. For this analysis we employed the R interface to the g:Profiler toolkit (gProfileR-v0.5 R package from the Comprehensive R Archive Network—CRAN). We focus our study on GO:BP functional evidence of the specific Sp1 signature, genes being sorted by their differential expression. For multiple testing correction the g:GOST native method, g:SCS, was applied. The electronic GO annotations were excluded from the analysis, and we used a 0.05 p-value threshold to select the significantly enriched GO terms. We also excluded terms containing less than 5 genes. To resume the gene list enrichement results the terms were grouped by a moderate hierarchical filtering (Table 1, S2 Table). R-2.14.2 and R-3.0.1 were used for these analyses.

**Table 1:** Functional analysis of Sp1 signature.

Term ID	Term name	p-value	Term size	Overlap size
GO:0044238	Primary metabolic process	1.11e-17	5444	379
GO:0044237	Cellular metabolic process	2.47e-15	5416	369
GO:0019222	Regulation of metabolic process	6.63e-11	3828	268
**GO:0045087**	**Innate immune response**	**3.84e-08**	**191**	**33**
GO:0035556	Intracellular signal transduction	2.28e-06	1382	112
GO:0009966	Regulation of signal transduction	4.54e-05	1643	122
GO:0051702	Interaction with symbiont	7.01e-05	39	11
GO:0016043	Cellular component organization	1.00e-04	3338	214
**GO:0035458**	**Cellular response to interferon-beta**	**1.00e-04**	**24**	**6**
GO:0034613	Cellular protein localization	2.54e-03	727	62
GO:0006886	Intracellular protein transport	6.96e-03	454	41
**GO:0060340**	**Positive regulation of type I interferon-mediated signaling pathway**	**7.84e-03**	**8**	**5**
GO:0044699	Single-organism process	8.63e-03	8909	475
**GO:0032606**	**Type I interferon production**	**9.86e-03**	**38**	**10**
GO:0010935	Regulation of macrophage cytokine production	4.33e-02	10	5
GO:0061082	Myeloid leukocyte cyokine production	4.46e-02	16	6

The gene list enrichment analysis from the Gene Ontology of the SP1 specific signature was performed with g:Profiler. The moderate hierarchical filtering used here allows a compact representation of gene list enrichment results. Significantly enriched GO terms containing less than 5 genes were excluded.

### Cell culture and reagents

The human embryonic kidney (HEK) 293T, mouse lymphoma EL4 and EL4 overexpressing the murine NKG2D ligand Rae (EL4-Rae), murine fibroblast 3T3 and the human lung epithelial cell line A549 cell lines were all cultured in Dulbecco’s modified Eagle’s medium (DMEM, Life Technologies) supplemented with 6% FCS (Lonza), 50μg/mL gentamicin, 2mM L-Glutamin and 10mM HEPES buffer (pH 7,4) (all form Life Technologies). The medium for mouse embryonic fibroblasts (MEFs) was supplemented with 100μM β-mercaptoethanol. The murine IL-3-dependent BaF3 cell line was maintained in complete medium supplemented with 5% IL-3 as described previously [30]. BaF3 clones expressing inducible Sp1 (full-length Sp1, BaF3-Sp1) and GFP were obtained as described [9, 14]. To repress ectopic Sp1 expression, cells were grown in presence of doxycycline (dox) (30ng/ml, Sigma). To induce Sp1 and GFP expression, cells were washed 3 times and cultured without dox (Tetracycline-off system). To induce BiP expression or XBP1 splicing in BaF3, cells were incubated 12h with Tunicamycin (TM) (10μg/ml, Sigma). To induce MDA5 and RIG-I expression in A549 cells, cells were incubated 16 h with human IFNa (1000units/ml, Merck, a gift from N. Goutagny). Rae overexpression in EL4-Rae was followed by flow cytometry using anti-mRae Ab (1:200, Clone 186107, R&D systems).

### Retroviral transductions and cell purification

Bicistronic retroviruses coding for CD2 marker alone (pMIC), CD2 followed by full-length human Sp1 (Sp1) or CD2 followed by mutated Sp1 Zn^2,3^ (mutations of the second and third Zn Fingers) (Zn). CD2 marker allow transduced cells identification as previously described [9, 14]. Retroviral particles were generated in 293T HEK packaging cells by transfection with Sp1, Sp1 Zn^2,3^ (Zn), or empty vector (pMIC) plasmid and concentrated with sucrose gradient separation procedure. Retroviral transduction was performed in 6-well plates, and selection of CD2 transduced cells was released at indicated time by magnetic selection using autoMACS technology (Miltenyi Biotec), according to the manufacturer’s protocol. Briefly, transduced cells were incubated 30 min with 4μg/ml PE-conjugated monoclonal Ab against murine CD2 (BD Biosciences) in Phosphate-buffered saline (PBS) containing 0,5% Bovine Serum Albumin (BSA) and 2mM EDTA. Cells were labelled with anti-PE microbeads (Miltenyi Biotec) and CD2 positive cells were magnetically selected by passing through an autoMACS (Miltenyi Biotec). The purity of positive fractions was evaluated by flow cytometry and was routinely above 95%.

### Mice

BALB/c mice were purchased from Charles River Laboratories and housed under specific pathogen-free conditions in our animal facility “Plateau de Biologie Expérimentale de la Souris” (AniRA-PBES). *In vivo* experimental procedures and the experimental use of primary mouse embryonic fibroblasts were approved by our local ethics committee (CECCAPP, Comite d’Evaluation Commun au Centre Leon Berard, à l’Animalerie de transit de l’ENS, au PBES et au laboratoire P4), and accreditations have been obtained from governmental agencies.

### RNA extraction, quantitative PCR and XBP1 splicing assay

Total RNA was prepared with RNeasy kit (Qiagen) and cDNA was synthetized with iScript cDNA Synthesis Kit (Bio-Rad). For qPCR experiments on MEFs, specific retrotranscription and preamplification steps were performed. Specific retrotranscription was made using 2μL of pooled reverse primers (2 pmole of each primer), 2μL of RNA, 1 μL of dNTP mix (10 mM). This mix was heated for 5 min at 65°C. Then, 4μL of 5x first strand buffer, 2μL of DTT (0,1M) and 1μL of RNase out (40 UI/ μL, Invitrogen) were added to the mix and incubated at 42°C for 2 min. 1μL of SuperScript II Reverse Transcriptase (Invitrogen) was added and the mix was incubated at 42°C for 50 min and 15 min at 70°C. Obtained cDNA were then preamplified with Fastart Universal SYBR GreenMaster (Roche, #04913850001) and specific primers (0,5μM). All preamplification reactions were cycled using the following parameters: 2 min at 95°C, 20 2-step cycles composed of 30 sec at 95°C and 4 min at 60°C. Each of preamplified sample was 1/10 diluted and qPCR was performed. For small RNAs (<200-nucleotides) preparation, the *mirVana* miRNA Isolation *Kit* (Invitrogen) was used, according to manufacturer’s protocol. Quantitative PCR was carried out using Platinum SYBRGreen qPCR SuperMix-UDG (Invitrogen) on ABI Prism applied 7000, StepOnePlus, or Taqman Low Density Array (TLDA) technology on 7900HT Fast Real-Time PCR System, according to manufacturer’s protocol (Applied Biosystems). The relative amount of mRNA was calculated by the comparative threshold cycle method with HPRT or GAPDH house keeping genes as control. Primer sequences used are detailed in S3 Table. The XBP1-splicing assay was performed using Iwakoshi and colleagues protocol [31]. Primers encompassing the spliced sequence in XBP1 mRNA (5′-ACACGCTTGGGAATGGACAC-3′ and 5′-CCATGGGAAGATGTTCTGGG-3′) were used for PCR amplification, and products were separated by electrophoresis through a 2.5% agarose gel and visualized by SYBRSafe staining (Invitrogen).

### Luciferase reporter assays

Transient transfection of HEK293T cells was performed using the calcium-phosphate precipitation method as previously described [32]. pCR3 empty vector and pCR3-Cardif-flag were gifts from J. Tschopp. Luciferase reporter assays were performed according to the manufacturer’s instructions (Promega).

### Immunoblot analysis

Cells were washed in cold PBS and resuspended in lysis buffer (5mM Hepes pH7.4, 150mM NaCl, 1% Nonidet P-40, 5mM EDTA, 10% glycérol and complete protease inhibitor cocktail (Roche)) for 20 min on ice. Equal amounts of proteins were loaded on NuPAGE 4–12% or 10% and transferred on nitrocellulose membrane using iBlot gel transfer stacks (all from Life Technologies). Abs used for immunoblot analysis were polyclonal rabbit antiserum Sp1 (home made; [9]), anti-caspase-3 (Cell signaling, #9661), anti-MDA5 (clone AT113, Enzo Life Sciences), anti-actine (Sigma), anti-BIP/GRP78 (BD transduction laboratories, #610979), anti-IRF7 (Abcam, #ab62505), anti-RIG-I (clone D14G6 XP) and anti-ISG-15 (#2743) from Cell Signaling. All Abs used were diluted at 1:1000, except anti-caspase-3 (1:2000), anti-BIP (1:500) and anti-ISG15 (1:200).

### Immunofluorescence microscopy

BaF3-Sp1 cells were treated for 24 h for H2AX staining and 20 h for RIG-I staining in the presence or absence of dox. As positive control, BaF3 cells were treated with etoposide (2μg/ml, Sigma) for 24 h. Cells were then seeded on polylysine-coated 12-mm glass coverslips, fixed with 4% paraformaldehyde (Thermoscientific) in PBS and permeabilized with 0,25% Triton X-100 (BDH) in PBS. Specimens were then incubated for 120 min with 1:50 anti-RIG-I Ab (clone C:15) (Santa Cruz biotechnologies) followed by 30 min with Dylight-549 conjugated anti-goat Ab (Jackson Immunoresearch), or with 1:200 anti-phospho H2A.X (clone JBW301, Millipore) followed by 1:250 biotin anti-mouse Ab (Caltag) and streptavidin-TRITC to study phospho-H2A.X foci formation. Slides were mounted with ProLong antifade (Invitrogen). Images of cells were obtained using a Zeiss AxioImager microscope and analyzed on ImageJ software.

### Chemotaxis assays

For *in vitro* chemotaxis assays, neutrophils were purified from BALB/c mice bone-marrow, using PE-conjugated anti-Ly6G Ab (BD Biosciences) and autoMACS technology (Miltenyi Biotec), according to manufacturer’s protocol. 5x10^5^ purified neutrophils were incubated for 3 h in transwell chambers with 5μm polycarbonate filters (Costar) in the presence of BaF3-Sp1 inducible clone previously treated (Ctrl) or not (Sp1) with dox for 24 h. Transmigrated neutrophils Ly6G^+^ were counted by flow cytometry. For *in vivo* chemotaxis assay, BaF3-Sp1 inducible cells were cultured 18 h in absence of dox and injected in the peritoneal cavity of BALB/c mice. PBS injected mice were used as controls. Six hours after injection, peritoneal washes were performed, cells were harvested and stained with anti-Ly6G (eBioscience) and anti-CD11b (BD Biosciences) Abs. All surface stainings were performed at 4°C in the dark in PBS supplemented with 1% FCS and 0,09% NaN3 (Sigma-Aldrich). Cellular recruitment was evaluated by flow cytometry.

### ELISA

ELISA of mouse CXCL-4 was performed using R&D systems kit (#DY595), according to the manufacturer’s protocol.

### Flow cytometry

Flow cytometry experiments weree performed on FACSCanto or LSR II instruments (BD Biosciences) and analyzed with FlowJo Software (TreeStar).

### Statistical analysis

Statistical analysis were run on Prism5 (GraphPad Software).

## Results

### Sp1 overexpression in untransformed cells is not associated with DNA damage or ER stress

**Fig 1:**
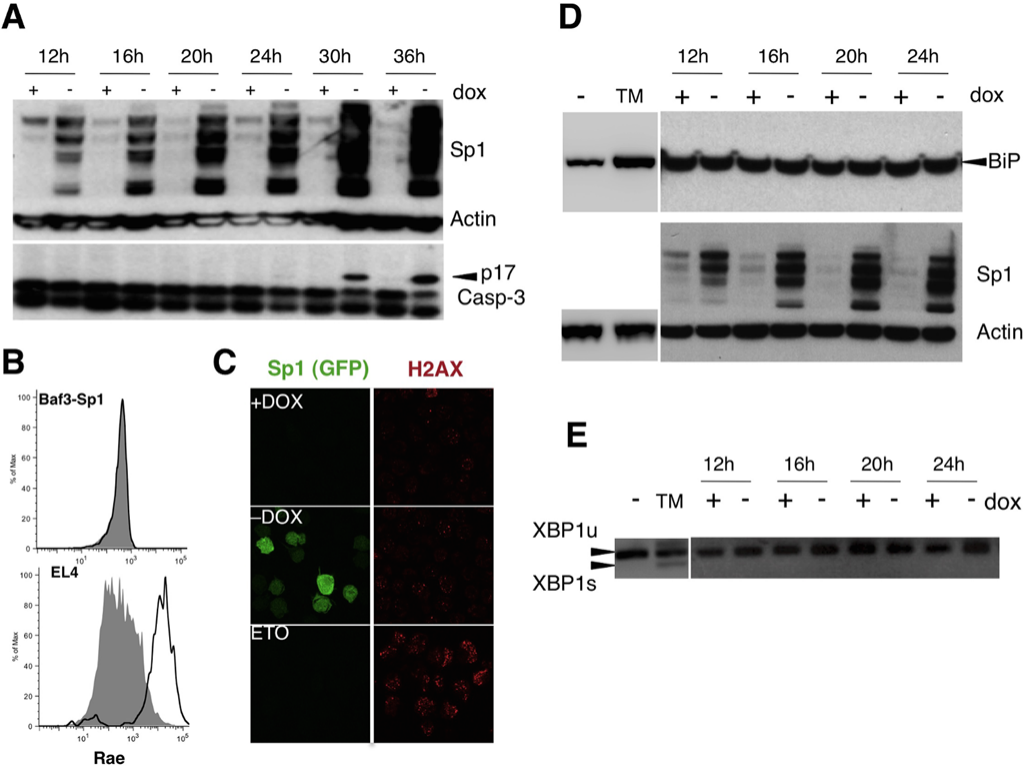
Characterization of the early cellular response upon Sp1 overexpression. BaF3-Sp1 cells were grown in the presence or absence of dox (Tetracycline-off system to induce Sp1 expression). (A) Cell extracts were collected at the indicated time and expression of Sp1, caspase-3 and actin was analyzed by immunoblot. (B) Twenty hours after dox withdrawal, Rae expression was evaluated by flow cytometry (+DOX: shaded histogram,-DOX: plain line). EL4 (shaded histogram) and EL4-Rae (plain line) cells were used as controls. (C) H2AX staining was performed on BaF3-Sp1 cells grown 24 h in the presence (+DOX) or absence (-DOX) of dox. BaF3 cells treated 24 h with etoposide (2μg/ml) were used as positive control (ETO). (D) Cell extracts were collected at the indicated times and expression of Sp1, BiP and actin was revealed by immunoblot. BaF3 cells treated 12 h with Tunicamycin (TM) (10μg/ml) were used as positive control for BIP expression. (E) RNA extracts were prepared at the indicated times and XBP1 splicing was evaluated by PCR. BaF3 cells treated 12 h with TM (10μg/ml) were used as positive control. Data are representative of at least two independent experiments.

We previously demonstrated that the overexpression of Sp1 induced an inhibition of cell cycle progression [14], followed by apoptotic cell death [9] in untransformed cells. In BaF3 clones generated earlier in the laboratory expressing inducible Sp1 and GFP (named BaF3-Sp1) [9], cell cycle slowdown is detected 22 h after Sp1 induction [14]. Apoptosis starts 30 h after Sp1 induction as demonstrated by caspase-3 cleavage (Fig. 1A). However, Sp1 expression is detected at the protein level as soon as 12 h after induction (Fig. 1A). We therefore wondered whether Sp1 overexpression may be associated to an early intrinsic response. Sp1 phosphorylation is induced by DNA damage, and Sp1 accumulation at damaged sites suggested a potential role in the DNA repair pathway [33]. So we first investigated whether increased Sp1 level may lead to the activation of a DNA damage response in BaF3-Sp1. We looked at the expression of the NKG2D ligand Rae as a marker of DNA damage response [34], but no up-regulation was detected in BaF3-Sp1 upon induction of exogenous Sp1 expression following doxycycline (dox) removal (Fig. 1B). In parallel, we also looked at phospho-H2A.X foci formation which is a classical hallmark of DNA damage response [35], and results show that increased Sp1 level does not induce phospho-H2A.X foci formation in BaF3 cells, whereas DNA damaging agent etoposide treatment does (Fig. 1C). The results clearly suggested that Sp1 overexpression does not lead to a DNA damage response. We next asked whether ER stress may be activated early after Sp1 induction. We first looked at Grp78/BiP protein expression as an ER stress marker [36] in BaF3-Sp1 cells upon dox removal, but BiP was never up-regulated by western blot. In contrast, positive control BaF3 cells treated with an ER stress inducer, Tunicamycin (TM), up-regulated BiP (Fig. 1D), demonstrating indeed these cells have intact ER stress response. We also studied the splicing of the transcription factor XBP1, the downstream target of the ER stress sensor kinase IRE1alpha, occuring during ER stress [37] by specific RT-PCR, and no mature form of XBP1 was detected upon Sp1 induction in BaF3-Sp1 cells in contrast to positive control BaF3 cells treated with TM (Fig. 1E). All these results demonstrated that increased Sp1 level is not associated with the induction of a DNA damage response or with an ER stress responses at early time points.

### Sp1 overexpression activates an innate immune transcriptome

To identify the early response activated by Sp1 overexpression, a functional analysis of microarrays data previously generated ([14]; ArrayExpress accession number E-MEXP-1702) was performed with the g:Profiler method. We first refined the list of genes over-expressed from a fold change (FC)>1.3 to a FC>1.5 (1187 genes, FC> = 1.5; S1 Table) and this more specific list signature was used for functional enrichment analysis via the g:Profiler toolkit for Biological Process subontology of Gene Ontology (GO:BP) [28, 29]. g:Profiler presents two major advantages for studies implying long gene lists as ours: a custom multiple testing correction procedure and the analysis of ordered gene lists (see details in Materials and Methods section). Differentially expressed genes following Sp1 overexpression belonged to 176 GO:BP significantly enriched terms corresponding to 16 more general enriched terms (Table 1, S2 Table) such as metabolism related terms, cell death (“single-organism process”) and more surprisingly “innate immune response”. Additionnaly, among the 16 more general enriched terms, three are directly connected to the “innate immun response” term: “cellular response to interferon-beta”, “positive regulation of type I interferon-mediated signaling pathway”, and “type I interferon production”. We finally performed a Gene Set Enrichment Analysis (GSEA) (http://www.broadinstitute.org/gsea; [38, 39]) on our microarray dataset of deregulated genes by Sp1 (FC> = 1.5; S1 Table) and we found that 30% of the first 20 enriched gene sets are related to innate immune response. All these results clearly demonstrated that increased Sp1 level in untransformed cells is associated with the activation of an innate immune transcriptome.

### Sp1 overexpression leads to the activation of the antiviral RIG-I pathway

**Fig 2:**
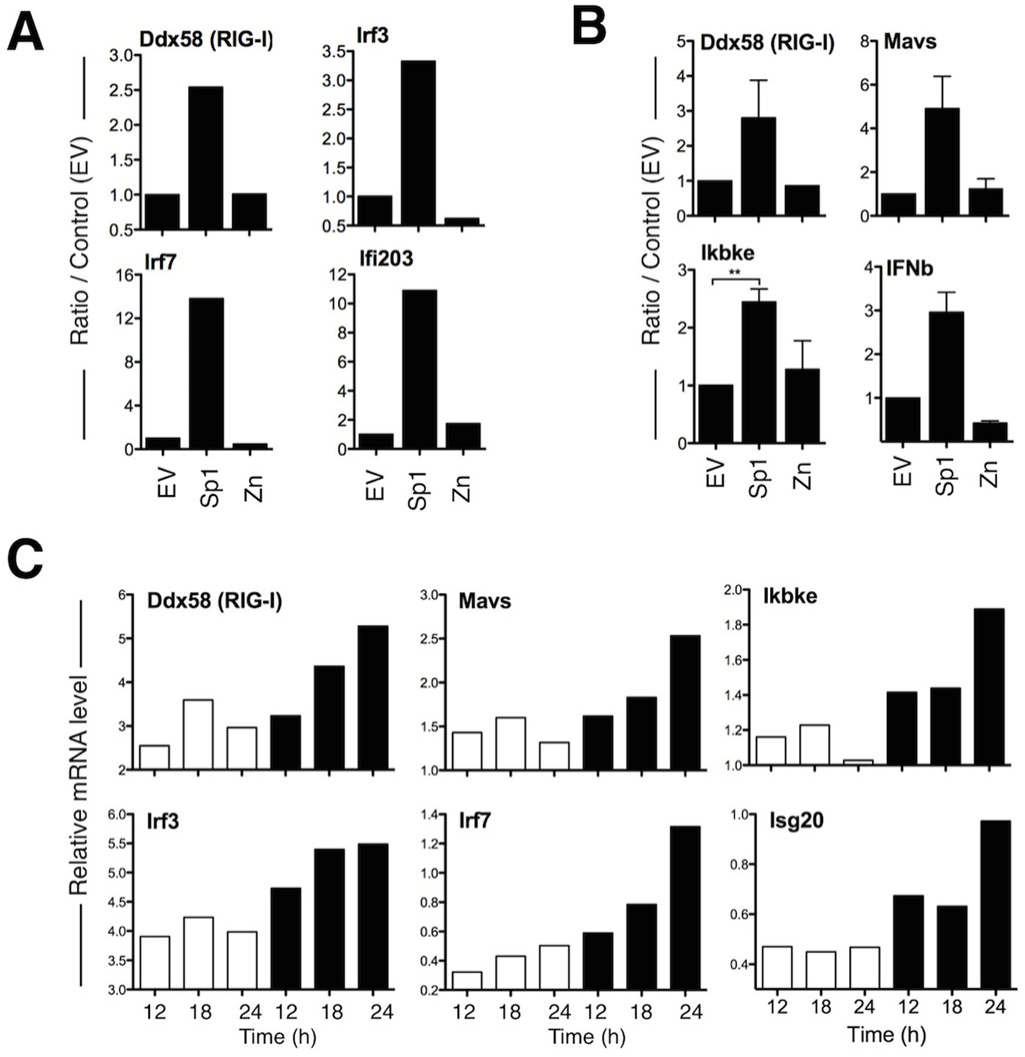
Sp1 overexpression activates genes of the RIG-I pathway. (A-B) BaF3 (A) and 3T3 (B) cells were transduced with retroviruses encoding wild-type Sp1, Sp1 Zn^2,3^ mutant (Zn) that does not bind DNA, or empty vector (EV). Transduced cells (CD2 positive) were purified by magnetic selection 30 h (BaF3) or 72 h (3T3) later, and mRNA levels of indicated genes were measured by RT-qPCR (TLDA used in B) and normalized to housekeeping genes (HPRT in A and Rps-21 in B) mRNA levels. Data are representative of one out of three independent experiments in A. In B panel, data are representative of two independent experiments and statistical analysis was performed using 2-tailed *t* tests. Levels of significance are expressed as follows: **P <0.01. (C) BaF3-Sp1 inducible cell line was grown with (white bars) or without (black bars) dox for indicated times, and relative mRNA levels of indicated genes were quantified by RT-qPCR (TLDA). Data are representative of one out of four independent experiments.

**Fig 3:**
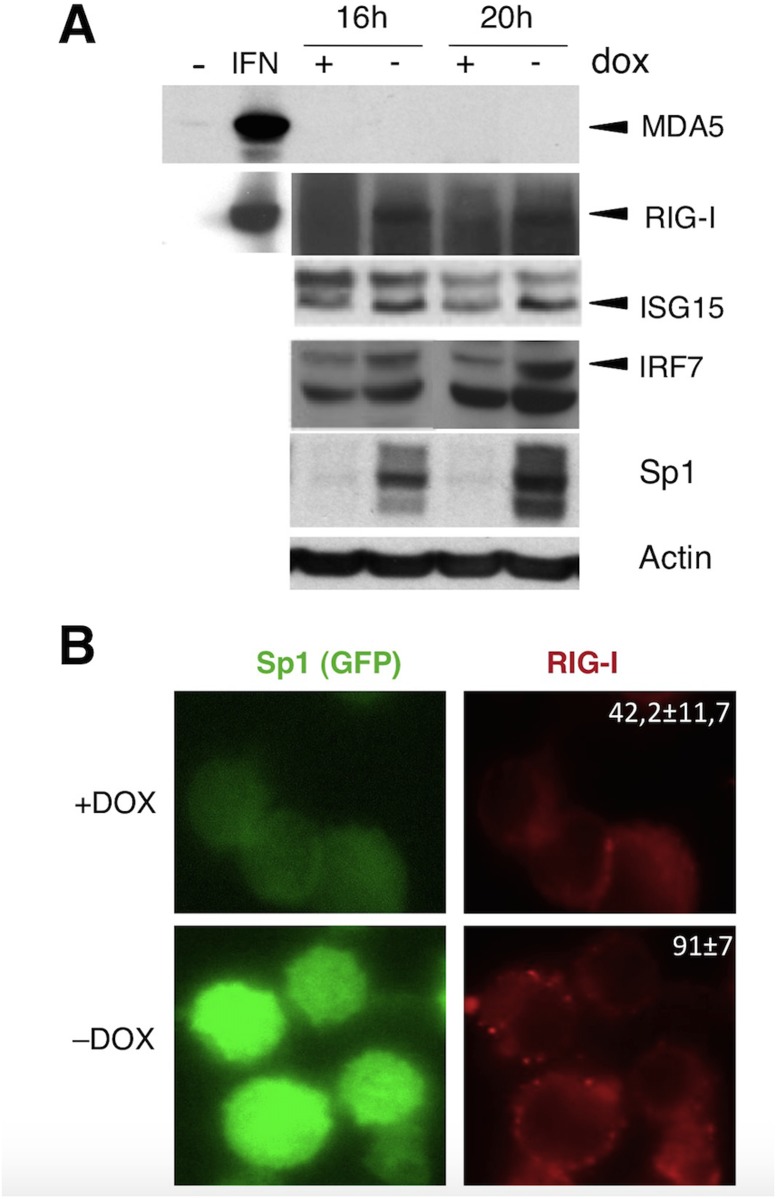
Proteins of the antiviral RIG-I pathway are induced by increased Sp1 level. BaF3-Sp1 cells were grown in the presence or absence of dox. (A) Cell extracts were collected at indicated time and expression of the indicated proteins was analyzed by immunoblot. A549 cells treated 16 h with IFNa (1000U/ml) were used as positive control (IFN) for MDA5 and RIG-I induction. (B) BaF3-Sp1 cells were cultured for 20 h, fixed and stained for RIG-I (red) and analyzed by fluorescent microscopy. The percentage of cells presenting RIG-I punctuated staining is indicated, and corresponds to the mean of 8 independent acquisitions. Data are representative of one out of two independent experiments.

The analysis of genes of the innate immune transcriptome induced by Sp1 overexpression leads to the identification of IFN inducible genes as well as genes implicated in the antiviral RIG-I pathway. The gene list is detailed in S2 Table. We first validated a set of genes using real-time quantitative PCR (qPCR) or Taqman Low Density Array technology (TLDA) in murine BaF3 cell line and 3T3 fibroblasts transduced with Sp1. In such untransformed cells, Sp1-induced apoptosis occured but the kinetic is slower than in the BaF3-Sp1 inducible system [9]. Confirming the microarray data, increased Sp1 level induces in both cell types the expression of essential components of the RIG-I signaling pathway such as the sensor *Ddx58*, the adaptor *Mavs*, the kinase *Ikbke* (IKKe), transcription factors *Irf3* and *Irf7* as well as the ISG genes *Ifi203* and *Ifnb* (Fig. 2A and 2B), indicating that this response may be activated in immune and non-immune cells. This gene activation is dependent on the capacity of Sp1 to bind DNA as a mutated form of Sp1 that does not bind DNA, Sp1 Zn^2,3^ mutant (Zn) [14], does not induce the upregulation of these genes (Fig. 2A and 2B). We next studied the RIG-I pathway activation in the inducible system BaF3-Sp1. As shown in Fig. 2C, genes belonging to the RIG-I pathway are activated as soon as 12 or 18 h after Sp1 induction, demonstrating that the antiviral RIG-I pathway is an early response induced by increased Sp1 level, before Sp1-mediated cell cycle slowdown (detected between 22 and 28 h of culture [14]) and apoptosis (starting at 30 h of culture, Fig. 1A). To examine whether the activation of the RIG-I pathway transcriptome is associated to an up-regulation at the protein level, we next evaluated the induction of RIG-I, ISG15 and IRF7 by western blot using the inducible Sp1 expression system BaF3-Sp1. The results show the induction of these proteins as soon as 16 h after dox removal (Fig. 3A). In contrast MDA5 is not detected (Fig. 3A), suggesting specific activation of the RIG-I sensor by Sp1. Finally, we looked at RIG-I activation by fluorescence microscopy and we observed an enhanced punctuated staining of RIG-I upon Sp1 induction (-DOX: 91±7% vs +DOX: 42,2±11,7%) (Fig. 3B), suggesting the formation of RIG-I aggregates as it was described for MAVS activation in a context of viral infection [40].

### Increased Sp1 level activates the OAS-RNAse L pathway and the production of small self-RNAs

**Fig 4:**
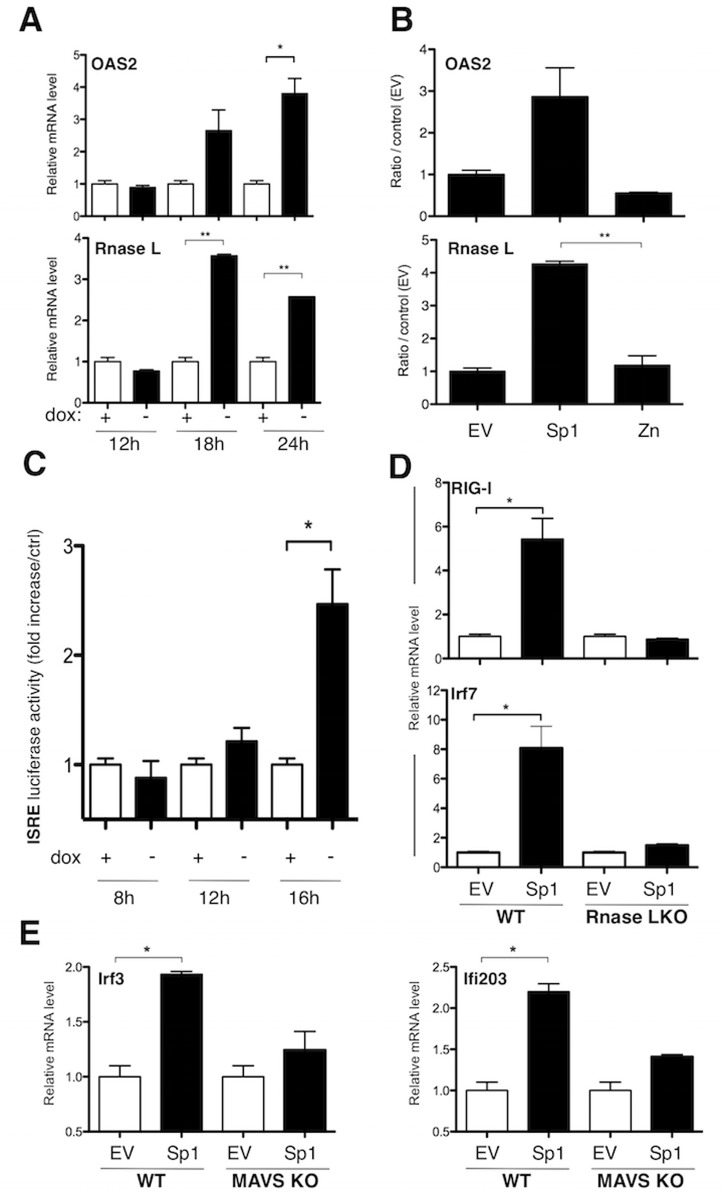
Sp1 activates the OAS-RNAse L pathway and the production of small self-RNAs. (A) BaF3-Sp1 inducible cell line was grown with (white bars) or without (black bars) dox for the indicated times, and relative mRNA levels of OAS2 and RNase L genes were quantified by RT-qPCR. (B, D, E) 3T3 (B) and MEFs (D, E) WT or deficient for the RNase L (RNase L KO) or MAVS (MAVS KO) were transduced with Sp1, Sp1 Zn^2,3^ (Zn) or empty vector (EV). Transduced cells (CD2 positive) were purified 72 h post-transduction by magnetic selection and relative mRNA levels of indicated genes were quantified by RT-qPCR. (C) Small RNAs from BaF3-Sp1 cells following induction Sp1 or not were extracted at the indicated times, and transfected into 293HEK cells (5μg per condition). Luciferase reporter assay was performed to analyze the ISRE promoter activity. Data are mean values ± standard deviation (SD) from one experiment representative of two or three independent experiments. Statistical analysis were performed using 2-tailed *t* tests. Levels of significance are expressed as follows: *P <0.05; **P <0.01.

We demonstrated that the capacity of Sp1 to bind DNA is necessary for the early activation of the RIG-I signaling pathway (Fig. 2A and 2B). We next wanted to know whether this activation is a direct consequence of Sp1-dependent gene transcription. A previous promoter analysis suggested that many up-regulated genes by Sp1 overexpression were not direct transcriptional targets of Sp1 [14]. We used another bioinformatic tool, oPOSSUM (http://www.cisreg.ca/oPOSSUM/; [41]), to look for Sp1 binding sites enrichment in our list of deregulated genes by Sp1 (FC> = 1.5; S1 Table) and in a list corresponding to “RIG-I-like receptor signaling pathway” in the KEGG pathway database (http://www.genome.jp/kegg/). No enrichment for Sp1 binding sites was observed in either of the lists. Finally, we tested whether overexpression of Sp1 in human embryonic kidney (HEK) 293T cells may directly induce specific promoter activity of specific genes activated downstream of RIG-I, such as the IFNβ-responsive promoter, the IRF3-dependent interferon-stimulated regulatory element (ISRE) or the NF-kB-dependent promoter. Overexpression of Sp1 or the mutated form Sp1 Zn^2,3^ (Zn) do not activate these promoters, whereas MAVS (positive control) does (data not shown). All these results strongly suggested that the activation of the RIG-I pathway is an indirect event induced by Sp1 overexpression. A major study highlighted that small self-RNAs (<200-nucleotides) generated by the 2’, 5’-oligoadenylate synthetase (OAS)-RNase L endoribonuclease pathway amplify antiviral innate responses mediated by RIG-I, MDA5 and MAVS [42]. We therefore hypothesized that a similar OAS-RNase L pathway may be activated upon Sp1 overexpression, initiating the activation of the antiviral RIG-I pathway. We first showed that *Oas2* and *Rnasel* genes were both up-regulated after Sp1 overexpression (FC> = 1.5, S1 Table). This expression was independant of endogenous mSp1 (data not shown). We validated by qPCR the early up-regulation of both genes in BaF3-Sp1 and 3T3 cells. As shown in Fig. 4A and 4B, both genes were up-regulated 18 h after dox removal in the inducible BaF3-Sp1 and day 3 post-transduction in 3T3 cells. To determine whether small self-RNAs may be generated upon increased Sp1 level, we purified total RNAs and small RNAs (<200-nucleotides) from BaF3-Sp1 cells. Hence, Sp1 overexpressing or control BaF3 cells were harvested at different time points, and then total RNAs and small RNAs were extracted and purified. No quantitative difference in total RNAs and small RNAs was measured between both conditions. We next transfected the same quantity of RNAs in 293T cells overexpressing an ISRE-dependent promoter. As shown in Fig. 4C, we observed an increase of the ISRE luciferase activity by small RNAs as soon as 12 h after Sp1 induction in BaF3-Sp1 cells, whereas total RNAs never activate the IRF3 promoter. These results strongly suggest that small self-RNAs, but not total RNAs, generated upon increased Sp1 level are able to activate the IRF3 promoter. Finally, to firmly demonstrate that the OAS-RNase L-RIG-I pathway is mandatory for intrinsic innate immune response activated by Sp1overexpression, we studied by qPCR the induction of *Ddx58* (RIG-I), *Irf7*, *Irf3 or Ifi203* in primary mouse embryonic fibroblasts (MEFs) wild type (WT) or deficient for RNase L or the RLR adaptor MAVS. As shown in Fig. 4D and 4E, indicated genes were up-regulated in primary MEFs WT upon Sp1 overexpression with the same kinetics than in 3T3 fibroblasts, and this expression is abolished in MEFs deficient for RNase L or MAVS. In conclusion, Sp1 overexpression in untransformed cells leads to the early activation of the OAS-RNase L-RIG-I pathway and to the generation of small self-RNAs that are able to activate IRF3 promoter activity.

### Cells overexpressing Sp1 produce inflammatory chemokine CXCL4 and lead to immune cells recruitment

**Fig 5:**
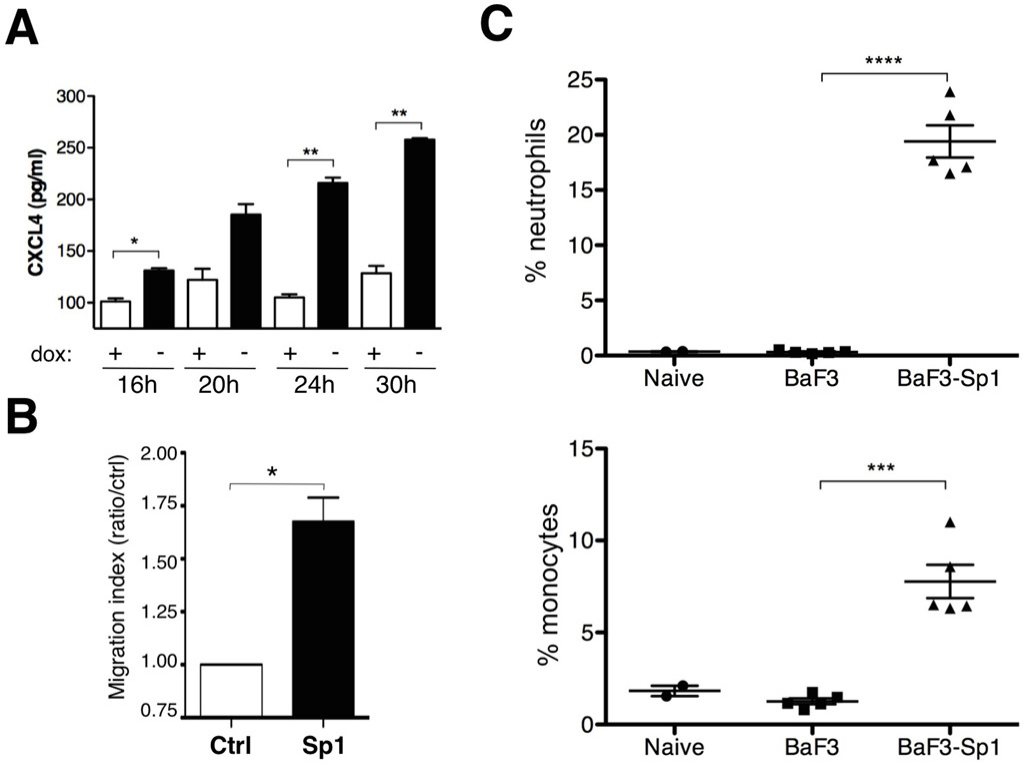
Immune cells are recruited by cells overexpressing Sp1. (A) BaF3-Sp1 cells were grown in the presence (+) or absence (-) of dox. CXCL4 production by BaF3-Sp1 was measured at the indicated times by ELISA. (B) BaF3-Sp1 cells were treated with or without dox for 24 h and incubated in the presence of neutrophils into transwell chambers during 3 h. Neutrophils recruitment (Ly6G^+^ CD11b^+^ cells) was assessed by flow cytometry. (C) BaF3 and BaF3-Sp1 cells were grown 18 h in absence or presence of dox and injected intra-peritoneally into mice. PBS injected mice were used as controls (Naive). Peritoneal washes were performed 6 h after injection. Cells were then harvested and cellular recruitment (CD11b^+^ Ly6G^-^ cells) was estimated by flow cytometry. These results are from one representative out of three independent experiments. Statistical analysis were performed using 2-tailed *t* tests. Levels of significance are expressed as follows: *P <0.05; **P <0.01; ***P = 0.0001; ****P <0.0001.

Evidences suggested that senescence, a cellular response that blocks proliferation, activates a self secretory network including inflammatory IL-6 and IL-8 [43], and CXCR2-binding chemokines [44]. Moreover, it was recently demonstrated that RIG-I signaling is involved in the senesence-associated secretory phenotype (SASP) in senescent cells, leading to the expression of inflammatory cytokines [45]. We therefore asked whether Sp1-induced RIG-I activation might lead to the production of inflammatory cytokines. Interestingly, the second most up-regulated gene upon Sp1 overexpression in BaF3 cells (list FC> = 1.5, S1 Table) corresponds to Platelet factor 4 (Pf4, also known as CXCL4), a potent chemotactic factor for neutrophils and monocytes already highlighted in our previous study [14]. We tested whether BaF3-Sp1 cells are able to secrete such chemokine and as shown in Fig. 5A, CXCL4 is secreted by BaF3-Sp1 cells after 16 h of dox removal, and continuously increases with time of culture. Moreover, using a chemotaxis assay, we showed that Sp1 overexpression in BaF3 cells is associated to an enhanced recruitment of neutrophils *in vitro* (Fig. 5B). We finally asked whether cells overexpressing Sp1 might also recruit phagocytes *in vivo*. BaF3 or BaF3-Sp1 inducible clones were cultured without dox for 18 h *in vitro* and then injected intraperitoneally into mice. Six hours later, the recruitment in the peritoneum of neutrophils (Ly6G^+^ CD11b^+^) and monocytes (CD11b^+^ Ly6G^-^) was evaluated by flow cytometry. As shown in Fig. 5C, BaF3-Sp1 injected mice showed abundant recruitment of neutrophils in their abdominal cavities, which was significantly higher than mice that received BaF3 cells. A similar enhanced influx of monocytes into the peritoneum of BaF3-Sp1 injected mice was observed (Fig. 5C). Altogether, these results showed that the intrinsic cellular response induced by Sp1 overexpression is associated to the secretion of chemotactic mediators including CXCL4, allowing the recruitment of inflammatory cells.

## Discussion

**Fig 6:**
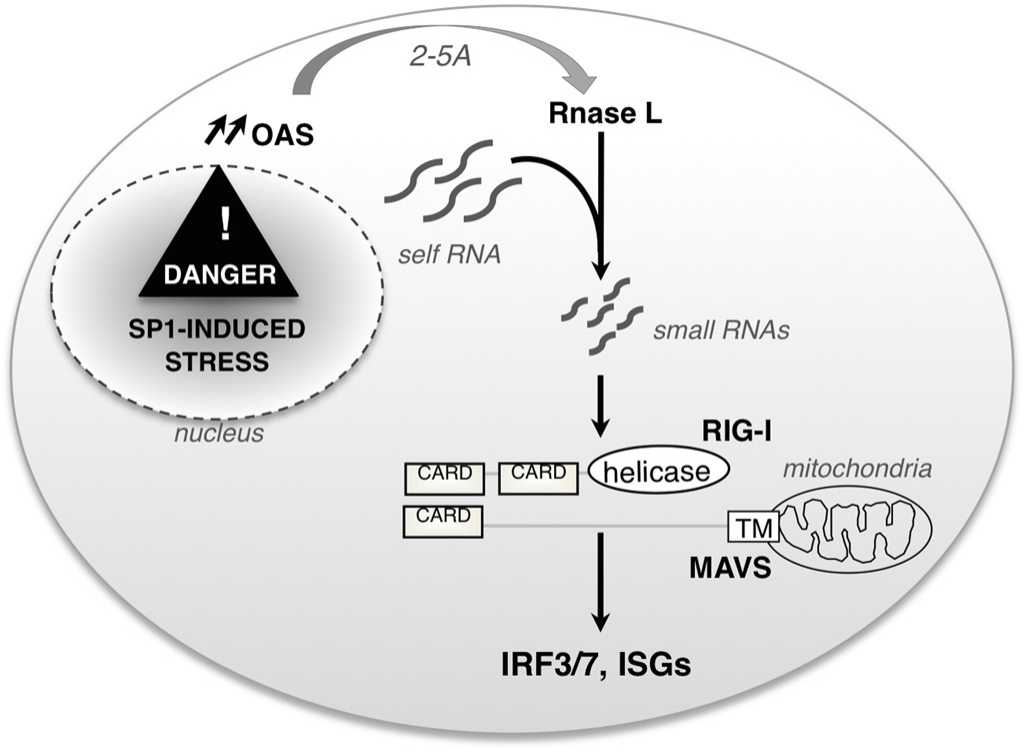
A schematic overview of the signaling pathway triggered by increased Sp1 level. Upon Sp1 overexpression, *Oas2* and *Rnasel* genes are upregulated and small self-RNAs are produced. Sensing of small self-RNAs leads to the activation of the sensor RIG-I, the IRF3/7 transcription factors and downstream effector targets such as ISGs.

Sp1 is upregulated in several tumor tissues, suggesting a role in tumor maintenance [46]. The level of Sp1 has been also shown to strongly increase during early stage of cellular transformation [15, 16], but the role of such overexpression is not known. We have previously documented that increasing Sp1 level in untransformed cells induces a cell cycle slowdown [14] that is followed by apoptosis [9]. Using a microarray approach, we observed that increased Sp1 level turns on an early innate immune transcriptome that precedes cell cycle slow-down and apopotosis. This early intrinsic response is characterized by the induction of the OAS-RNase L axis and the generation of small self-RNAs, leading to the activation of the antiviral RIG-I pathway (Fig. 6).

Sp1 is a transcription factor playing a central role during development [6] and in multiple cellular processes, such as cell cycle progression [14], DNA damage [11] or apoptosis [8–10]. The apoptosis induced by Sp1 overexpression is p53-dependent [8] and involves caspase-3 activation (Fig. 1A; [9]), but the signaling pathway leading to the activation of apoptosis is not fully characterized. Here we showed that Sp1 activates the antiviral RIG-I signaling pathway. The role of the RIG-I pathway in the regulation of cell death has been suggested by several studies. For example, RIG-I and MDA5 were identified as initiators of a proapoptotic signaling pathway requiring the BH3-only protein Noxa in melanoma cells [47]. Moreover, overexpression of the adaptor MAVS in 293T cells leads to caspase-3 activation [48]. Finally, recent reports have shown that MAVS plays a major role in virus-induced apoptosis, as for example by inducing caspase-3 activation upon Vesicular Stomatitis Virus (VSV) infection [49]. Therefore, the RIG-I pathway activation upon increased Sp1 level may be involved in Sp1-induced apoptotic cascade upstream of caspase-3 activation. However, further experiments would need to be performed to confirm this hypothesis.

Because Sp1-binding sites are found in the promoters of some viral genes, several studies have suggested a role of Sp1 in the transactivation of viral genes [50, 51] and in viral replication [52]. However, from the perspective of the host immune response, little is know about Sp1 role in the regulation of antiviral responses. Our results clearly highlighted an unknown function of Sp1 as a regulator of the RIG-I antiviral pathway. A pioneering aspect of our work is the activation of the OAS-RNase L axis by increased Sp1 level. The amplification of the antiviral RIG-I pathway by RNAse L was initially identified by Malathi and colleagues in the context of a viral infection [42], but our data are the first demonstration, to our knowledge, that the activation of the OAS-RNase L axis may occur in the absence of pathogen. A recent study showed that Sp1 knockdown in human keratinocytes enhances viral replication by downregulating gene expression of the double-stranded RNA-dependent protein kinase (PKR) and OAS2 [52], suggesting that Sp1 regulates their transcription. Therefore, our results are in agreement with these data, as Sp1 overexpression enhances *Oas2* gene expression. Nevertheless, we never detected *Pkr* gene up-regulation upon Sp1 overexpression (S1 Table), suggesting that the antiviral pathway activated may be cell type-specific.

In our study, we demonstrated that increased Sp1 level induces an endogenous response that is not associated to a DNA damage response nor ER stress, but activates the OAS-RNase L axis and the production of small self-RNAs. We have clearly demonstrated that the induction of the RIG-I sensor is dependent of RNase L, and that small self-RNAs produced upon increased Sp1 level activate the IRF3 promoter, but the mechanism of OAS2 activation remains to be established. In human, there are four genes coding for OAS1, OAS2, OAS3 and OASL proteins. The OAS1 to-3 proteins have a characteristic polymerase activity, the 2’-5’ oligoadenylate (2–5A) synthetase activity, that is activated by double-stranded (ds)RNA in the context of a viral infection. 2–5As produced by OAS proteins then bind and activate the RNase L, leading to the degradation of cellular and viral RNA, and thus to the inhibition of protein synthesis and viral replication [53]. Sp1 has been suggested to control chromatin structure [54], and many Sp1 binding sites are localised next to noncoding RNA genes [4]. As Sp1 knockdown is associated to the downregulation of OAS2 gene expression, and that three putative Sp1 binding sites have been found in the OAS2 gene promoter [52], we may hypothezise that Sp1 overexpression leads to the direct upregulation of OAS2 and in parallel to the production of an excess of noncoding RNA. The upregulation of OAS2 then activates the RNase L that use cellular noncoding RNA as substrate to synthezise small self-RNAs that can serve as RIG-I ligands [42]. However, we cannot exclude that the activation of OAS2 is not a direct consequence of Sp1 transcriptional activity, but that Sp1 overexpression induces the production of cellular dsRNA that activate the OAS protein. Finally, we purified small self-RNAs generated upon Sp1 overexpression on a size criterion (less than 200-nucleotides), in order to get the same type of RNA known to amplify the antiviral RIG-I pathway in a viral context [42], but their exact nature is not known.

Transformation is associated with the capacity to bypass cellular checkpoints that control essential cellular pathways. The cellular perturbation induced by increased Sp1 level could represent a new barrier at the transcriptional level that cells would have to bypass to become transformed. As the ER stress response plays the role of quality control of translation, the OAS-RNase L axis would play the role of quality control at the transcriptional level. And the RIG-I sensor, firstly identified to detect exogenous viral RNA, may be activated by specific endogenous small self-RNAs generated at early stage of cellular transformation. Further experiments will be needed in order to confirm that Sp1-mediated inflammatory gene expression is relevant in the context of a tumor microenvironment.

## Supporting Information

S1 Table Microarray analysis: the Sp1 signature. Microarray generation and analysis of gene expression (FC> = 1.3) upon Sp1 overexpression was previously described [14]. A more specific Sp1 signature (FC> = 1.5, 1187 unique genes) was defined using “affy” and “limma” Bioconductor/R packages (details in Materials and Methods section) and presented here.
(PDF)

S2 Table Functional analysis of Sp1 signature. The gene list enrichment analysis from the Gene Ontology of the SP1 specific signature was performed with g:Profiler. The moderate hierarchical filtering used here allows a compact representation of gene list enrichment results. Significantly enriched GO terms containing less than 5 genes were excluded.
(PDF)

S3 Table Sequences of qPCR primers. (A) Primers used in Fig. 3, designed by our team. Primers for GAPDH, IFNβ and IRF7-b were used on StepOne+ Real-Time PCR Systems. Primers for HPRT, hSp1, RIG-I and IRF7-a were used on ABI PRISM 7000 Sequence Detection System. Ifi203, IFNz, IRF3, Jun and mSp1 were used on both systems. (B) Primers mix designed and validated by Qiagen, used in Fig. 5A and B. The catalog references are indicated.
(TIFF)
