# Supplementary material for: Overexpression of the Transcription Factor Sp1 Activates the OAS-RNAse L-RIG-I Pathway
Source: PLoS One. 2015 Mar 4;10(3):e0118551. doi: 10.1371/journal.pone.0118551 (PMC4349862; doi:10.1371/journal.pone.0118551)
Supplement: S1 Table — Microarray generation and analysis of gene expression (FC> = 1.3) upon Sp1 overexpression was previously described [14]. A more specific Sp1 signature (FC> = 1.5, 1187 unique genes) was defined using “affy” and “limma” Bioconductor/R packages (details in Materials and Methods section) and presented here. (PDF) [file pone.0118551.s001.pdf]

S1 Table. Microarray analysis: the Sp1 signature.

Gene Symbol	Gene Name	FC (SP1 vs Ctrl)	Entrez ID
Neo1	neogenin	11,22	18007
Pf4	platelet factor 4	8,99	56744
Stxbp6	syntaxin binding protein 6 (amisyn)	8,20	217517
Gbp1	guanylate binding protein 1	7,70	14468
Tmem51	transmembrane protein 51	7,09	214359
E230032D23Rik	RIKEN cDNA E230032D23 gene	7,04	414103
Pik3r3	phosphatidylinositol 3 kinase, regulatory subunit, polypeptide 3 (p55)	6,83	18710
Gsn	gelsolin	6,61	227753
Tmem71	transmembrane protein 71	6,61	213068
Arl6ip1	ADP-ribosylation factor-like 6 interacting protein 1	5,88	54208
Upp1	uridine phosphorylase 1	5,38	22271
Madcam1	mucosal vascular addressin cell adhesion molecule 1	5,30	17123
Gbp2	guanylate binding protein 2	5,27	14469
Arih1	ariadne ubiquitin-conjugating enzyme E2 binding protein homolog 1 (Drosophila)	5,25	23806
Siglec5	sialic acid binding Ig-like lectin 5	4,86	233186
Serpinf1	serine (or cysteine) peptidase inhibitor, clade F, member 1	4,86	20317
Rnasek	ribonuclease, RNase K	4,29	52898
Cd74	CD74 antigen (invariant polypeptide of major histocompatibility complex, class II antigen-associated)	4,18	16149
Jun	Jun oncogene	4,08	16476
Tmem173	transmembrane protein 173	4,07	72512
4933411B09Rik	RIKEN cDNA 4933411B09 gene	4,02	71123
St8sia4	ST8 alpha-N-acetyl-neuraminide alpha-2,8-sialyltransferase 4	3,96	20452
Irf7	interferon regulatory factor 7	3,84	54123
0610010O12Rik	RIKEN cDNA 0610010O12 gene	3,74	66060
Fam129a	family with sequence similarity 129, member A	3,74	63913
Tnfrsf9	tumor necrosis factor receptor superfamily, member 9	3,72	21942
Ccdc19	coiled-coil domain containing 19	3,71	71870
Calml4	calmodulin-like 4	3,69	75600
Lats2	large tumor suppressor 2	3,67	50523
Bmf	BCL2 modifying factor	3,61	171543
4930513N10Rik	RIKEN cDNA 4930513N10 gene	3,56	319960
Galnt3	UDP-N-acetyl-alpha-D-galactosamine:polypeptide N-acetylgalactosaminyltransferase 3	3,48	14425
Gbp3	guanylate binding protein 3	3,38	55932
Rgs2	regulator of G-protein signaling 2	3,34	19735
Slc11a1	solute carrier family 11 (proton-coupled divalent metal ion transporters), member 1	3,30	18173
Cnr2	cannabinoid receptor 2 (macrophage)	3,29	12802

Slc39a8	solute carrier family 39 (metal ion transporter), member 8	3,25	67547
Btg2	B-cell translocation gene 2, anti-proliferative	3,22	12227
Tgm2	transglutaminase 2, C polypeptide	3,17	21817
Prickle1	prickle homolog 1 (Drosophila)	3,17	106042
Ddc	dopa decarboxylase	3,15	13195
A630052C17Rik	RIKEN cDNA A630052C17 gene	3,15	320757
Eps8	epidermal growth factor receptor pathway substrate 8	3,14	13860
Cdkl2	cyclin-dependent kinase-like 2 (CDC2-related kinase)	3,13	53886
Tcp11l2	t-complex 11 (mouse) like 2	3,10	216198
Actg2	actin, gamma 2, smooth muscle, enteric	3,06	11468
Stap2	signal transducing adaptor family member 2	3,04	106766
9530077C05Rik	RIKEN cDNA 9530077C05 gene	3,03	68283
Vwa5a	von Willebrand factor A domain containing 5A	3,03	67776
Rbms1	RNA binding motif, single stranded interacting protein 1	3,00	56878
Myl4	myosin, light polypeptide 4	2,99	17896
LOC545086	hypothetical protein LOC545086	2,97	545086
Ifi203	interferon activated gene 203	2,94	15950
Tssk6	testis-specific serine kinase 6	2,94	83984
Aurkc	aurora kinase C	2,93	20871
Usp2	ubiquitin specific peptidase 2	2,89	53376
Parp3	poly (ADP-ribose) polymerase family, member 3	2,89	235587
Trp53inp2	transformation related protein 53 inducible nuclear protein 2	2,87	68728
Tmc4	transmembrane channel-like gene family 4	2,85	353499
Ckmt1	creatine kinase, mitochondrial 1, ubiquitous	2,83	12716
Mlana	melan-A	2,80	77836
Ptgr1	prostaglandin reductase 1	2,79	67103
Spata6	spermatogenesis associated 6	2,78	67946
4930524J08Rik	RIKEN cDNA 4930524J08 gene	2,78	75129
Muc1	mucin 1, transmembrane	2,76	17829
Klhl24	kelch-like 24 (Drosophila)	2,76	75785
Arid5a	AT rich interactive domain 5A (MRF1-like)	2,75	214855
Egr2	early growth response 2	2,73	13654
S100a11	S100 calcium binding protein A11 (calgizzarin)	2,72	20195
Gch1	GTP cyclohydrolase 1	2,70	14528
Parp14	poly (ADP-ribose) polymerase family, member 14	2,70	547253
Mecp2	methyl CpG binding protein 2	-2,68	17257
Padi3	peptidyl arginine deiminase, type III	2,67	18601
Gm5547	predicted gene 5547	2,66	433637
Gm11627	predicted gene 11627	2,66	#########
A130040M12Rik	RIKEN cDNA A130040M12 gene	2,65	319269
Cideb	cell death-inducing DNA fragmentation factor, alpha subunit-like effector B	2,64	12684
2610109H07Rik	RIKEN cDNA 2610109H07 gene	2,62	70433
Dppa2	developmental pluripotency associated 2	2,60	73703
5730414N17Rik	RIKEN cDNA 5730414N17 gene	2,57	70524
Anxa9	annexin A9	2,56	71790
Tspan13	tetraspanin 13	2,56	66109
Gm16515	predicted gene, Gm16515	2,55	24083
Ccrl2	chemokine (C-C motif) receptor-like 2	2,55	54199

Sgpl1	sphingosine phosphate lyase 1	2,54	20397
Pxmp2	peroxisomal membrane protein 2	2,51	19301
Abca1	ATP-binding cassette, sub-family A (ABC1), member 1	2,50	11303
Slfn5	schlafen 5	2,48	327978
Fos	FBJ osteosarcoma oncogene	2,48	14281
D7Wsu130e	DNA segment, Chr 7, Wayne State University 130, expressed	2,46	28017
Fam102a	family with sequence similarity 102, member A	2,46	98952
Asb11	ankyrin repeat and SOCS box-containing 11	2,45	68854
B230312A22Rik	RIKEN cDNA B230312A22 gene	2,45	230088
Gramd1c	GRAM domain containing 1C	2,45	207798
Sgk3	serum/glucocorticoid regulated kinase 3	2,44	170755
P2ry14	purinergic receptor P2Y, G-protein coupled, 14	2,42	140795
Abhd15	abhydrolase domain containing 15	2,41	67477
Rab11fip2	RAB11 family interacting protein 2 (class I)	-2,40	74998
Oasl2	2'-5' oligoadenylate synthetase-like 2	2,40	23962
Myeov2	myeloma overexpressed 2	2,40	66915
Sp1	trans-acting transcription factor 1	-2,39	20683
5430401H09Rik	RIKEN cDNA 5430401H09 gene	2,38	71396
Jhdm1d	jumonji C domain-containing histone demethylase 1 homolog D (S. cerevisiae)	2,38	338523
Clec2d	C-type lectin domain family 2, member d	-2,38	93694
Gm15698	transcription elongation factor B (SIII), polypeptide 2 pseudogene	2,37	217066
Spint1	serine protease inhibitor, Kunitz type 1	2,37	20732
Zfp943	zinc finger prtoein 943	2,37	74670
Rnd2	Rho family GTPase 2	2,37	11858
Gm9776	predicted gene 9776	2,37	328309
6030498E09Rik	RIKEN cDNA 6030498E09 gene	2,36	77883
Pcyt1b	phosphate cytidylyltransferase 1, choline, beta isoform	2,35	236899
Gmeb1	glucocorticoid modulatory element binding protein 1	-2,34	56809
Traf1	TNF receptor-associated factor 1	2,34	22029
Ccdc69	coiled-coil domain containing 69	2,33	52570
Scoc	short coiled-coil protein	-2,32	56367
Sfmbt1	Scm-like with four mbt domains 1	2,31	54650
Plekha4	pleckstrin homology domain containing, family A (phosphoinositide binding specific) member 4	2,30	69217
Klf6	Kruppel-like factor 6	2,30	23849
Ddx58	DEAD (Asp-Glu-Ala-Asp) box polypeptide 58	2,30	230073
Pja2	praja 2, RING-H2 motif containing	-2,29	224938
Cpeb4	cytoplasmic polyadenylation element binding protein 4	2,29	67579
Gvin1	GTPase, very large interferon inducible 1	2,29	74558
C3	complement component 3	2,28	12266
Gm7111	predicted gene 7111	2,28	633385
Lsp1	lymphocyte specific 1	2,28	16985
LOC664787	similar to Sp110 nuclear body protein	2,28	664787
Zfp119b	zinc finger protein 119b	2,27	240120
E2f2	E2F transcription factor 2	2,27	242705
Trpv2	transient receptor potential cation channel, subfamily V, member 2	2,27	22368
Ypel2	yippee-like 2 (Drosophila)	2,27	77864

Dusp1	dual specificity phosphatase 1	2,27	19252
Ctss	cathepsin S	2,26	13040
Lyz1	lysozyme 1	2,26	17110
Gnptg	N-acetylglucosamine-1-phosphotransferase, gamma subunit	2,26	214505
Rell1	RELT-like 1	2,25	100532
Sepp1	selenoprotein P, plasma, 1	2,24	20363
Jdp2	Jun dimerization protein 2	2,24	81703
Cela1	chymotrypsin-like elastase family, member 1	2,24	109901
Trp53inp1	transformation related protein 53 inducible nuclear protein 1	2,24	60599
Atp6v1d	ATPase, H+ transporting, lysosomal V1 subunit D	2,23	73834
Mr1	major histocompatibility complex, class I-related	2,23	15064
Specc1	sperm antigen with calponin homology and coiled-coil domains 1	2,22	432572
Plvap	plasmalemma vesicle associated protein	2,22	84094
Malat1	metastasis associated lung adenocarcinoma transcript 1 (non-coding RNA)	2,22	72289
Pdp1	pyruvate dehyrogenase phosphatase catalytic subunit 1	-2,22	381511
2610002M06Rik	RIKEN cDNA 2610002M06 gene	-2,22	67028
Cnga1	cyclic nucleotide gated channel alpha 1	2,22	12788
1110032A03Rik	RIKEN cDNA 1110032A03 gene	2,21	68721
A930001N09Rik	RIKEN cDNA A930001N09 gene	2,21	77128
Map1lc3b	microtubule-associated protein 1 light chain 3 beta	2,21	67443
Ramp1	receptor (calcitonin) activity modifying protein 1	2,21	51801
Ncf1	neutrophil cytosolic factor 1	2,21	17969
Ppp1r15a	protein phosphatase 1, regulatory (inhibitor) subunit 15A	2,20	17872
Dock5	dedicator of cytokinesis 5	-2,19	68813
Tubb2a	tubulin, beta 2A	2,19	22151
Gemin4	gem (nuclear organelle) associated protein 4	-2,19	276919
Ppp3cb	protein phosphatase 3, catalytic subunit, beta isoform	-2,19	19056
Casp4	caspase 4, apoptosis-related cysteine peptidase	2,18	12363
Trim9	tripartite motif-containing 9	2,18	94090
1700008O03Rik	RIKEN cDNA 1700008O03 gene	2,17	69349
Dcaf11	DDB1 and CUL4 associated factor 11	2,17	28199
Cenpj	centromere protein J	2,17	219103
Lpar6	lysophosphatidic acid receptor 6	2,17	67168
Slc14a1	solute carrier family 14 (urea transporter), member 1	-2,17	108052
Wsb1	WD repeat and SOCS box-containing 1	2,17	78889
A430035B10Rik	RIKEN cDNA A430035B10 gene	2,17	320312
Mmp9	matrix metallopeptidase 9	2,16	17395
Carhsp1	calcium regulated heat stable protein 1	2,16	52502
Tnfrsf23	tumor necrosis factor receptor superfamily, member 23	2,16	79201
Rwdd3	RWD domain containing 3	-2,16	66568
Fgf23	fibroblast growth factor 23	-2,16	64654
Morc3	microrchidia 3	-2,15	338467
Cebpd	CCAAT/enhancer binding protein (C/EBP), delta	2,15	12609
D17H6S56E-5	DNA segment, Chr 17, human D6S56E 5	2,15	110956
Crem	cAMP responsive element modulator	2,14	12916
Rasip1	Ras interacting protein 1	2,14	69903

Chrnb1	cholinergic receptor, nicotinic, beta polypeptide 1 (muscle)	2,13	11443
Itfg3	integrin alpha FG-GAP repeat containing 3	2,13	106581
Tapbpl	TAP binding protein-like	2,12	213233
Zrsr1	zinc finger (CCCH type), RNA binding motif and serine/arginine rich 1	2,12	22183
Itsn1	intersectin 1 (SH3 domain protein 1A)	2,12	16443
Csrnp2	cysteine-serine-rich nuclear protein 2	2,12	207785
Cd79b	CD79B antigen	2,11	15985
Fuca2	fucosidase, alpha-L- 2, plasma	2,11	66848
Ugdh	UDP-glucose dehydrogenase	2,11	22235
Cd82	CD82 antigen	2,10	12521
Isg20	interferon-stimulated protein	2,10	57444
Fez2	fasciculation and elongation protein zeta 2 (zygin II)	-2,09	225020
Zfp828	zinc finger protein 828	-2,08	101994
Dgka	diacylglycerol kinase, alpha	2,08	13139
Rab7l1	RAB7, member RAS oncogene family-like 1	2,08	226422
Mtap	methylthioadenosine phosphorylase	-2,08	66902
Hmox1	heme oxygenase (decycling) 1	2,08	15368
Gm19827	predicted gene, 19827	2,08	#########
Cldnd1	claudin domain containing 1	-2,06	224250
Gpx1	glutathione peroxidase 1	2,06	14775
Dynlt3	dynein light chain Tctex-type 3	-2,05	67117
Gstt1	glutathione S-transferase, theta 1	2,05	14871
Dpp7	dipeptidylpeptidase 7	2,04	83768
Zfp639	zinc finger protein 639	-2,04	67778
BC022687	cDNA sequence BC022687	2,04	217887
Rnasel	ribonuclease L (2', 5'-oligoisoadenylate synthetase-dependent)	2,04	24014
Zkscan4	zinc finger with KRAB and SCAN domains 4	2,04	544922
BC048403	cDNA sequence BC048403	-2,03	270802
1700024h08rik	RIKEN cDNA 1700024H08 gene	2,03	#########
Sin3a	transcriptional regulator, SIN3A (yeast)	-2,03	20466
Herc6	hect domain and RLD 6	2,03	67138
Acpl2	acid phosphatase-like 2	2,03	235534
Klc4	kinesin light chain 4	2,03	74764
Ccdc82	coiled-coil domain containing 82	-2,03	66396
Hist1h2bc	histone cluster 1, H2bc	2,03	68024
Gm14005	predicted gene 14005	2,03	#########
Ulbp1	UL16 binding protein 1	2,03	77777
Npl	N-acetylneuraminate pyruvate lyase	2,03	74091
Cdkn2c	cyclin-dependent kinase inhibitor 2C (p18, inhibits CDK4)	2,02	12580
Fam149b	family with sequence similarity 149, member B	-2,02	105428
Abat	4-aminobutyrate aminotransferase	2,02	268860
Zfp758	zinc finger protein 758	2,02	224598
Fhod3	formin homology 2 domain containing 3	2,01	225288
Pus7l	pseudouridylate synthase 7 homolog (S. cerevisiae)-like	-2,01	78895
4930558N01Rik	RIKEN cDNA 4930558N01 gene	2,01	75840
Ifit1	interferon-induced protein with tetratricopeptide repeats 1	2,01	15957
Slco2b1	solute carrier organic anion transporter family, member 2b1	2,01	101488

Plin2	perilipin 2	2,01	11520
Nlrp10	NLR family, pyrin domain containing 10	2,01	244202
Erbb3	v-erb-b2 erythroblastic leukemia viral oncogene homolog 3 (avian)	2,01	13867
Phospho2	phosphatase, orphan 2	2,01	73373
Lrrc42	leucine rich repeat containing 42	-2,00	77809
Nln	neurolysin (metallopeptidase M3 family)	-2,00	75805
4930515G01Rik	RIKEN cDNA 4930515G01 gene	2,00	67642
0610031J06Rik	RIKEN cDNA 0610031J06 gene	2,00	56700
Akr1b10	aldo-keto reductase family 1, member B10 (aldose reductase)	2,00	67861
Ccng2	cyclin G2	2,00	12452
Prmt7	protein arginine N-methyltransferase 7	1,99	214572
Rbpms	RNA binding protein gene with multiple splicing	1,99	19663
1110002E22Rik	RIKEN cDNA 1110002E22 gene	1,99	#########
Ppox	protoporphyrinogen oxidase	1,99	19044
2310047M10Rik	RIKEN cDNA 2310047M10 gene	1,99	71923
Zfp36	zinc finger protein 36	1,99	22695
St7l	suppression of tumorigenicity 7-like	1,99	229681
Rfxap	regulatory factor X-associated protein	1,98	170767
Fam46a	family with sequence similarity 46, member A	1,98	212943
Rcor3	REST corepressor 3	-1,98	214742
Napsa	napsin A aspartic peptidase	1,98	16541
Gadd45a	growth arrest and DNA-damage-inducible 45 alpha	1,98	13197
Kdelr3	KDEL (Lys-Asp-Glu-Leu) endoplasmic reticulum protein retention receptor 3	1,98	105785
Gm16740	predicted gene, 16740	1,98	#########
Rab34	RAB34, member of RAS oncogene family	1,97	19376
Zbtb12	zinc finger and BTB domain containing 12	-1,97	193736
Ubr2	ubiquitin protein ligase E3 component n-recognin 2	1,97	224826
Rhpn2	rhophilin, Rho GTPase binding protein 2	1,97	52428
6330403M23Rik	RIKEN cDNA 6330403M23 gene	-1,97	109169
Epsti1	epithelial stromal interaction 1 (breast)	1,97	108670
Abcb1b	ATP-binding cassette, sub-family B (MDR/TAP), member 1B	1,97	18669
Fgd6	FYVE, RhoGEF and PH domain containing 6	1,97	13998
Renbp	renin binding protein	1,96	19703
Cep97	centrosomal protein 97	1,96	74201
Tmem50b	transmembrane protein 50B	1,96	77975
Cbfa2t3	core-binding factor, runt domain, alpha subunit 2, translocated to, 3 (human)	-1,96	12398
A630072M18Rik	RIKEN cDNA A630072M18 gene	1,96	320770
Zfp473	zinc finger protein 473	1,96	243963
Serac1	serine active site containing 1	-1,95	321007
Dub1	deubiquitinating enzyme 1	1,95	13531
Tgoln1	trans-golgi network protein	1,95	22134
9130230N09Rik	RIKEN cDNA 9130230N09 gene	1,95	#########
Tnni3	troponin I, cardiac 3	1,95	21954
Hivep3	human immunodeficiency virus type I enhancer binding protein 3	-1,95	16656
Ms4a6d	membrane-spanning 4-domains, subfamily A, member 6D	1,95	68774
Gp9	glycoprotein 9 (platelet)	1,94	54368
Neu1	neuraminidase 1	1,94	18010

2810002N01Rik	RIKEN cDNA 2810002N01 gene	1,94	68020
Clu	clusterin	1,94	12759
Stx11	syntaxin 11	1,94	74732
Trpc2	transient receptor potential cation channel, subfamily C, member 2	1,94	22064
Parp9	poly (ADP-ribose) polymerase family, member 9	1,94	80285
St3gal4	ST3 beta-galactoside alpha-2,3-sialyltransferase 4	-1,94	20443
Arsb	arylsulfatase B	1,93	11881
Gla	galactosidase, alpha	1,93	11605
Lgals3	lectin, galactose binding, soluble 3	1,93	16854
Bcl9	B-cell CLL/lymphoma 9	-1,93	77578
Dnajc21	DnaJ (Hsp40) homolog, subfamily C, member 21	-1,93	78244
Vpreb1	pre-B lymphocyte gene 1	1,92	22362
Ccdc102a	coiled-coil domain containing 102A	1,92	234582
Ccdc28a	coiled-coil domain containing 28A	1,92	215814
Ero1lb	ERO1-like beta (S. cerevisiae)	1,92	67475
Slc16a4	solute carrier family 16 (monocarboxylic acid transporters), member 4	1,92	229699
Fbxo4	F-box protein 4	1,92	106052
1110067D22Rik	RIKEN cDNA 1110067D22 gene	1,92	216551
Fbxo6	F-box protein 6	1,92	50762
A430108G06Rik	RIKEN cDNA A430108G06 gene	1,92	319650
Txndc16	thioredoxin domain containing 16	1,92	70561
9530028C05	hypothetical protein 9530028C05	1,92	330256
Gatsl2	GATS protein-like 2	1,92	80909
Ccpg1	cell cycle progression 1	1,91	72278
Rnf167	ring finger protein 167	1,91	70510
Pld3	phospholipase D family, member 3	1,91	18807
Ecm1	extracellular matrix protein 1	1,91	13601
Ccnd2	cyclin D2	-1,91	12444
Il2rb	interleukin 2 receptor, beta chain	1,91	16185
Ebi3	Epstein-Barr virus induced gene 3	1,91	50498
Esr1	estrogen receptor 1 (alpha)	1,91	13982
Mobkl2a	MOB1, Mps One Binder kinase activator-like 2A (yeast)	-1,91	208228
Trap1a	tumor rejection antigen P1A	1,91	22037
Nfkbie	nuclear factor of kappa light polypeptide gene enhancer in B-cells inhibitor, epsilon	1,91	18037
2610305J24Rik	RIKEN cDNA 2610305J24 gene	1,90	#########
Zfand2b	zinc finger, AN1 type domain 2B	-1,90	68818
Trf	transferrin	1,90	22041
Cln3	ceroid lipofuscinosis, neuronal 3, juvenile (Batten, Spielmeyer-Vogt disease)	1,90	12752
Ikzf4	IKAROS family zinc finger 4	1,90	22781
Zfp954	zinc finger protein 954	1,90	232853
Taf13	TAF13 RNA polymerase II, TATA box binding protein (TBP)-associated factor	1,90	99730
Mpg	N-methylpurine-DNA glycosylase	1,90	268395
Rnf114	ring finger protein 114	1,89	81018
A030001D16Rik	RIKEN cDNA A030001D16 gene	-1,89	399638
Tln1	talin 1	-1,89	21894
Prx	periaxin	1,89	19153
2610002J02Rik	RIKEN cDNA 2610002J02 gene	-1,89	67513
Cd53	CD53 antigen	1,89	12508

Megf9	multiple EGF-like-domains 9	-1,89	230316
2610044O15Rik	RIKEN cDNA 2610044O15 gene	1,88	72139
Lss	lanosterol synthase	-1,88	16987
Det1	de-etiolated homolog 1 (Arabidopsis)	-1,88	76375
Ypel5	yippee-like 5 (Drosophila)	1,88	383295
S100pbp	S100P binding protein	-1,88	74648
Dnase2a	deoxyribonuclease II alpha	1,88	13423
4922501C03Rik	RIKEN cDNA 4922501C03 gene	1,88	382090
Neurl3	neuralized homolog 3 homolog (Drosophila)	1,88	214854
4930524L23Rik	RIKEN cDNA 4930524L23 gene	1,88	78185
Qrich2	glutamine rich 2	1,88	217341
Qser1	glutamine and serine rich 1	-1,87	99003
Irgm1	immunity-related GTPase family M member 1	1,87	15944
L3mbtl3	l(3)mbt-like 3 (Drosophila)	1,87	237339
Zfp560	zinc finger protein 560	1,87	434377
Cnn2	calponin 2	1,87	12798
Tceanc	transcription elongation factor A (SII) N-terminal and central domain containing	-1,87	245695
Ahi1	Abelson helper integration site 1	1,87	52906
Hemgn	hemogen	1,87	93966
9530046B11Rik	RIKEN cDNA 9530046B11 gene	1,87	78421
Pdpk1	3-phosphoinositide dependent protein kinase 1	-1,86	18607
4930578N16Rik	RIKEN cDNA 4930578N16 gene	1,86	75051
Dusp16	dual specificity phosphatase 16	1,86	70686
Phc1	polyhomeotic-like 1 (Drosophila)	-1,86	13619
Sccpdh	saccharopine dehydrogenase (putative)	1,86	109232
Tpp1	tripeptidyl peptidase I	1,86	12751
Ctns	cystinosis, nephropathic	1,86	83429
Optn	optineurin	1,85	71648
Sp4	trans-acting transcription factor 4	-1,85	20688
Tmco4	transmembrane and coiled-coil domains 4	1,85	77056
Mfsd11	major facilitator superfamily domain containing 11	1,85	69900
Hspb11	heat shock protein family B (small), member 11	-1,85	72938
Pik3ca	phosphatidylinositol 3-kinase, catalytic, alpha polypeptide	-1,85	18706
Depdc1a	DEP domain containing 1a	1,85	76131
Gm608	predicted gene 608	-1,85	207806
Ccdc122	coiled-coil domain containing 122	1,85	108811
Atg12	autophagy-related 12 (yeast)	1,85	67526
Ffar2	free fatty acid receptor 2	1,85	233079
Suv420h2	suppressor of variegation 4-20 homolog 2 (Drosophila)	-1,85	232811
Mex3d	mex3 homolog D (C. elegans)	-1,85	237400
Trmt2b	TRM2 tRNA methyltransferase 2 homolog B (S. cerevisiae)	-1,85	215201
Runx2	runt related transcription factor 2	1,85	12393
Pnma5	paraneoplastic antigen family 5	1,85	385377
Ndufaf3	NADH dehydrogenase (ubiquinone) 1 alpha subcomplex, assembly factor 3	1,85	66706
Zfp280d	zinc finger protein 280D	-1,85	235469
Hsd17b11	hydroxysteroid (17-beta) dehydrogenase 11	1,85	114664
Sypl	synaptophysin-like protein	-1,85	19027
Pif1	PIF1 5'-to-3' DNA helicase homolog (S. cerevisiae)	1,84	208084
Dctd	dCMP deaminase	-1,84	320685

Grap2	GRB2-related adaptor protein 2	1,84	17444
Smcr8	Smith-Magenis syndrome chromosome region, candidate 8 homolog (human)	1,84	237782
Pink1	PTEN induced putative kinase 1	1,84	68943
Cenpk	centromere protein K	1,84	60411
2810416G20Rik	RIKEN cDNA 2810416G20 gene	-1,84	#########
Ier5	immediate early response 5	1,84	15939
Atf5	activating transcription factor 5	1,84	107503
Igf2bp2	insulin-like growth factor 2 mRNA binding protein 2	-1,84	319765
A930026I22Rik	RIKEN cDNA A930026I22 gene	1,83	77970
Slc18a1	solute carrier family 18 (vesicular monoamine), member 1	1,83	110877
BC031353	cDNA sequence BC031353	1,83	235493
2500004C02Rik	RIKEN cDNA 2500004C02 gene	1,83	72326
Zbp1	Z-DNA binding protein 1	1,83	58203
Trip4	thyroid hormone receptor interactor 4	1,83	56404
Tyrobp	TYRO protein tyrosine kinase binding protein	1,83	22177
Pxmp4	peroxisomal membrane protein 4	1,83	59038
Rhbdd1	rhomboid domain containing 1	1,83	76867
Sacs	sacsin	-1,83	50720
Adnp2	ADNP homeobox 2	-1,83	240442
Gtf2b	general transcription factor IIB	1,83	229906
Mtap4	microtubule-associated protein 4	-1,82	17758
Klhl17	kelch-like 17 (Drosophila)	-1,82	231003
Acta2	actin, alpha 2, smooth muscle, aorta	1,82	11475
Zfp748	zinc finger protein 748	-1,82	212276
Tcta	T-cell leukemia translocation altered gene	1,82	102791
Mad2l1bp	MAD2L1 binding protein	1,82	66591
Pbrm1	polybromo 1	-1,82	66923
Gpsm1	G-protein signalling modulator 1 (AGS3-like, C. elegans)	-1,82	67839
Zyg11b	zyg-II homolog B (C. elegans)	-1,82	414872
Hexim2	hexamethylene bis-acetamide inducible 2	1,82	71059
Spib	Spi-B transcription factor (Spi-1/PU.1 related)	1,82	272382
Tcrg-V1	T-cell receptor gamma, variable 1	-1,82	21632
Pik3ap1	phosphoinositide-3-kinase adaptor protein 1	1,82	83490
Slc38a9	solute carrier family 38, member 9	1,81	268706
Fam160b2	family with sequence similarity 160, member B2	-1,81	239170
Ppip5k1	diphosphoinositol pentakisphosphate kinase 1	-1,81	327655
Atp6v1e1	ATPase, H+ transporting, lysosomal V1 subunit E1	1,81	11973
1700071A11Rik	RIKEN cDNA 1700071A11 gene	-1,81	76611
Zfp935	zinc finger protein 935	1,81	71508
Tdrd7	tudor domain containing 7	1,81	100121
Zfp788	zinc finger protein 788	-1,81	67607
B3gnt5	UDP-GlcNAc:betaGal beta-1,3-N-acetylglucosaminyltransferase 5	-1,81	108105
Chaf1b	chromatin assembly factor 1, subunit B (p60)	1,81	110749
Parp6	poly (ADP-ribose) polymerase family, member 6	-1,81	67287
Armc8	armadillo repeat containing 8	-1,81	74125
Sema4a	sema domain, immunoglobulin domain (Ig), transmembrane domain (TM) and short cytoplasmic domain, (semaphorin) 4A	1,81	20351
4632427E13Rik	RIKEN cDNA 4632427E13 gene	1,81	666737
Gmpr2	guanosine monophosphate reductase 2	1,81	105446

Cotl1	coactosin-like 1 (Dictyostelium)	1,81	72042
Mnd1	meiotic nuclear divisions 1 homolog (S. cerevisiae)	-1,81	76915
Usp32	ubiquitin specific peptidase 32	-1,81	237898
Pbx2	pre B-cell leukemia transcription factor 2	-1,80	18515
Arl3	ADP-ribosylation factor-like 3	-1,80	56350
C030046G05	hypothetical protein C030046G05	1,80	327885
Sgcb	sarcoglycan, beta (dystrophin-associated glycoprotein)	-1,80	24051
Adprh	ADP-ribosylarginine hydrolase	-1,80	11544
Ndst2	N-deacetylase/N-sulfotransferase (heparan glucosaminyl) 2	1,80	17423
Dok3	docking protein 3	1,80	27261
Tapbp	TAP binding protein	1,80	21356
Zfp78	zinc finger protein 78	1,80	330463
Gm5544	predicted gene 5544	-1,80	433632
Cenpl	centromere protein L	1,80	70454
1110003F02Rik	RIKEN cDNA 1110003F02 gene	1,80	68466
Ifi27l2a	interferon, alpha-inducible protein 27 like 2A	1,80	76933
Copg2	coatomer protein complex, subunit gamma 2	-1,80	54160
Hspa4l	heat shock protein 4 like	-1,80	18415
1500002F19Rik	RIKEN cDNA 1500002F19 gene	1,80	76683
Myo1c	myosin IC	-1,80	17913
Pttg1ip	pituitary tumor-transforming 1 interacting protein	-1,79	108705
Macf1	microtubule-actin crosslinking factor 1	-1,79	11426
Arhgap39	Rho GTPase activating protein 39	-1,79	223666
Rab13	RAB13, member RAS oncogene family	1,79	68328
Phf20l1	PHD finger protein 20-like 1	1,79	239510
Rbl1	retinoblastoma-like 1 (p107)	-1,79	19650
Wipi1	WD repeat domain, phosphoinositide interacting 1	-1,79	52639
Map3k8	mitogen-activated protein kinase kinase kinase 8	1,79	26410
9430023L20Rik	RIKEN cDNA 9430023L20 gene	1,79	68118
Wdfy1	WD repeat and FYVE domain containing 1	1,79	69368
Gdpd1	glycerophosphodiester phosphodiesterase domain containing 1	1,79	66569
Nfe2	nuclear factor, erythroid derived 2	-1,79	18022
Styx	serine/threonine/tyrosine interaction protein	-1,79	56291
Cyb5d1	cytochrome b5 domain containing 1	1,79	327951
Zfyve1	zinc finger, FYVE domain containing 1	1,79	217695
Lamp2	lysosomal-associated membrane protein 2	1,79	16784
Fam69a	family with sequence similarity 69, member A	-1,79	67266
Zfp667	zinc finger protein 667	1,79	384763
Zmym2	zinc finger, MYM-type 2	-1,79	76007
F2rl2	coagulation factor II (thrombin) receptor-like 2	1,78	14064
Senp8	SUMO/sentrin specific peptidase 8	-1,78	71599
Zbtb25	zinc finger and BTB domain containing 25	-1,78	109929
Socs6	suppressor of cytokine signaling 6	-1,78	54607
Mvp	major vault protein	1,78	78388
Nhlrc1	NHL repeat containing 1	-1,78	105193
LOC100505088	hypothetical LOC100505088	1,78	#########
2610005L07Rik	cadherin 11 pseudogene	1,78	381598
1110019D14Rik	RIKEN cDNA 1110019D14 gene	1,78	76311
Trpm7	transient receptor potential cation channel, subfamily M, member 7	-1,78	58800
Gm11110	predicted gene 11110	-1,78	#########

Ntpcr	nucleoside-triphosphatase, cancer-related	-1,78	66566
Mxd1	MAX dimerization protein 1	1,78	17119
Elavl1	ELAV (embryonic lethal, abnormal vision, Drosophila)-like 1 (Hu antigen R)	1,78	15568
Flcn	folliculin	1,77	216805
Csad	cysteine sulfinic acid decarboxylase	1,77	246277
Rap1gap2	RAP1 GTPase activating protein 2	1,77	380711
Ankrd27	ankyrin repeat domain 27 (VPS9 domain)	-1,77	245886
Fam118b	family with sequence similarity 118, member B	1,77	109229
Plekhb1	pleckstrin homology domain containing, family B (evectins) member 1	1,77	27276
Mdfic	MyoD family inhibitor domain containing	1,77	16543
D4Ertd22e	DNA segment, Chr 4, ERATO Doi 22, expressed	-1,77	213491
Hmg20b	high mobility group 20 B	-1,77	15353
Rab37	RAB37, member of RAS oncogene family	-1,77	58222
Gm4117	predicted gene 4117	-1,77	#########
Scly	selenocysteine lyase	1,77	50880
Creb3l1	cAMP responsive element binding protein 3-like 1	1,76	26427
Cgrrf1	cell growth regulator with ring finger domain 1	1,76	68755
Col5a1	collagen, type V, alpha 1	-1,76	12831
Tubb1	tubulin, beta 1	1,76	545486
Nbr1	neighbor of Brca1 gene 1	1,76	17966
Actr1b	ARP1 actin-related protein 1 homolog B, centractin beta (yeast)	-1,76	226977
Cyth3	cytohesin 3	-1,76	19159
Edn3	endothelin 3	1,76	13616
Dnajc12	DnaJ (Hsp40) homolog, subfamily C, member 12	1,76	30045
Impact	imprinted and ancient	1,76	16210
Dnajb9	DnaJ (Hsp40) homolog, subfamily B, member 9	1,76	27362
Itgb3bp	integrin beta 3 binding protein (beta3-endonexin)	1,76	67733
Rab31	RAB31, member RAS oncogene family	1,75	106572
Otos	otospiralin	1,75	260301
Parp8	poly (ADP-ribose) polymerase family, member 8	1,75	52552
AA645497	expressed sequence AA645497	1,75	#########
Hgfac	hepatocyte growth factor activator	1,75	54426
Rpgr	retinitis pigmentosa GTPase regulator	-1,75	19893
Fblim1	filamin binding LIM protein 1	1,75	74202
Gnpda2	glucosamine-6-phosphate deaminase 2	1,75	67980
Gm10374	predicted gene 10374	1,75	#########
Nbeal1	neurobeachin like 1	-1,75	269198
Tdp1	tyrosyl-DNA phosphodiesterase 1	-1,75	104884
Gtf2f1	general transcription factor IIF, polypeptide 1	1,75	98053
Nfic	nuclear factor I/C	-1,75	18029
Tlcd1	TLC domain containing 1	-1,75	68385
Rpp40	ribonuclease P 40 subunit (human)	-1,75	208366
Slco4a1	solute carrier organic anion transporter family, member 4a1	-1,75	108115
Galk1	galactokinase 1	-1,75	14635
Wdr89	WD repeat domain 89	-1,74	72338
Sh3glb1	SH3-domain GRB2-like B1 (endophilin)	-1,74	54673
Ppargc1b	peroxisome proliferative activated receptor, gamma, coactivator 1 beta	-1,74	170826
Laptm5	lysosomal-associated protein transmembrane 5	1,74	16792

Pfkfb3	6-phosphofructo-2-kinase/fructose-2,6-biphosphatase 3	-1,74	170768
Gimap8	GTPase, IMAP family member 8	1,74	243374
Tspan8	tetraspanin 8	1,74	216350
Fam195b	family with sequence similarity 195, member B	-1,74	192173
Npepps	aminopeptidase puromycin sensitive	-1,74	19155
Hist2h2be	histone cluster 2, H2be	1,74	319190
Tuba8	tubulin, alpha 8	-1,74	53857
Slc43a2	solute carrier family 43, member 2	1,74	215113
4933424C08Rik	RIKEN cDNA 4933424C08 gene	1,74	71185
Krcc1	lysine-rich coiled-coil 1	-1,74	57896
Wdr8	WD repeat domain 8	-1,74	59002
Dusp10	dual specificity phosphatase 10	1,74	63953
Tmem199	transmembrane protein 199	1,74	195040
Gbp7	guanylate binding protein 7	1,73	229900
Nr1d2	nuclear receptor subfamily 1, group D, member 2	-1,73	353187
Arf6	ADP-ribosylation factor 6	-1,73	11845
Hvcn1	hydrogen voltage-gated channel 1	1,73	74096
BC024659	cDNA sequence BC024659	-1,73	108934
Zxdc	ZXD family zinc finger C	1,73	80292
Bcl2l11	BCL2-like 11 (apoptosis facilitator)	1,73	12125
Pip4k2b	phosphatidylinositol-5-phosphate 4-kinase, type II, beta	-1,73	108083
Camkk2	calcium/calmodulin-dependent protein kinase kinase 2, beta	-1,73	207565
Eps8l2	EPS8-like 2	1,73	98845
Pmm1	phosphomannomutase 1	-1,73	29858
Zfp566	zinc finger protein 566	-1,73	72556
Avl9	AVL9 homolog (S. cerevisiase)	1,73	78937
Fcho2	FCH domain only 2	1,73	218503
Zfp119a	zinc finger protein 119a	1,73	104349
Gda	guanine deaminase	-1,73	14544
Usp34	ubiquitin specific peptidase 34	-1,73	17847
Nagk	N-acetylglucosamine kinase	1,73	56174
Zkscan14	zinc finger with KRAB and SCAN domains 14	1,73	67235
Cyb561d2	cytochrome b-561 domain containing 2	1,73	56368
Trib3	tribbles homolog 3 (Drosophila)	1,73	228775
Lipa	lysosomal acid lipase A	-1,73	16889
AI597479	expressed sequence AI597479	-1,73	98404
A430005L14Rik	RIKEN cDNA A430005L14 gene	-1,72	97159
Ada	adenosine deaminase	1,72	11486
Ift46	intraflagellar transport 46 homolog (Chlamydomonas)	1,72	76568
Bbs4	Bardet-Biedl syndrome 4 (human)	-1,72	102774
Selp	selectin, platelet	-1,72	20344
Add2	adducin 2 (beta)	1,72	11519
Tsga10	testis specific 10	-1,72	211484
Chmp5	chromatin modifying protein 5	1,72	76959
Szt2	seizure threshold 2	-1,72	230676
Ninl	ninein-like	-1,72	78177
Senp7	SUMO1/sentrin specific peptidase 7	-1,72	66315
Mum1	melanoma associated antigen (mutated) 1	-1,71	68114
Gpd1l	glycerol-3-phosphate dehydrogenase 1-like	-1,71	333433
Amz2	archaelysin family metallopeptidase 2	-1,71	13929

Creb3	cAMP responsive element binding protein 3	1,71	12913
Ebpl	emopamil binding protein-like	-1,71	68177
6720401G13Rik	RIKEN cDNA 6720401G13 gene	1,71	103012
Dcaf4	DDB1 and CUL4 associated factor 4	-1,71	73828
Zfp771	zinc finger protein 771	-1,71	244216
Serpina3g	serine (or cysteine) peptidase inhibitor, clade A, member 3G	1,71	20715
Gipc2	GIPC PDZ domain containing family, member 2	1,71	54120
Vsig10	V-set and immunoglobulin domain containing 10	1,71	231668
Nfkbib	nuclear factor of kappa light polypeptide gene enhancer in B-cells inhibitor, beta	1,71	18036
Bpgm	2,3-bisphosphoglycerate mutase	1,71	12183
Prr13	proline rich 13	1,71	66151
Polr2h	polymerase (RNA) II (DNA directed) polypeptide H	-1,71	245841
Klhl23	kelch-like 23 (Drosophila)	1,70	277396
Bbs12	Bardet-Biedl syndrome 12 (human)	-1,70	241950
Fam108c	family with sequence similarity 108, member C	-1,70	70178
Tnnt1	troponin T1, skeletal, slow	1,70	21955
Zfp238	zinc finger protein 238	-1,70	30928
Atxn7l3	ataxin 7-like 3	-1,70	217218
C330018D20Rik	RIKEN cDNA C330018D20 gene	1,70	77422
1700066B19Rik	RIKEN cDNA 1700066B19 gene	1,70	73449
Ccdc38	coiled-coil domain containing 38	1,70	237465
Tlr2	toll-like receptor 2	1,70	24088
Pvr	poliovirus receptor	1,70	52118
2310004I24Rik	RIKEN cDNA 2310004I24 gene	-1,70	66358
Immp1l	IMP1 inner mitochondrial membrane peptidase-like (S. cerevisiae)	1,70	66541
Slc12a5	solute carrier family 12, member 5	1,70	57138
Aplp2	amyloid beta (A4) precursor-like protein 2	1,70	11804
Wdsub1	WD repeat, SAM and U-box domain containing 1	-1,70	72137
Gatad2b	GATA zinc finger domain containing 2B	-1,70	229542
BC068157	cDNA sequence BC068157	1,70	73072
Nsmce1	non-SMC element 1 homolog (S. cerevisiae)	1,70	67711
Tab2	TGF-beta activated kinase 1/MAP3K7 binding protein 2	-1,70	68652
4933426M11Rik	RIKEN cDNA 4933426M11 gene	1,70	217684
Cmtm6	CKLF-like MARVEL transmembrane domain containing 6	-1,70	67213
Armc7	armadillo repeat containing 7	1,70	276905
Tcrg-V4	T-cell receptor gamma, variable 4	-1,70	21638
2010106G01Rik	RIKEN cDNA 2010106G01 gene	-1,70	66552
Syt16	synaptotagmin XVI	1,69	238266
Pik3ip1	phosphoinositide-3-kinase interacting protein 1	1,69	216505
Cdkl4	cyclin-dependent kinase-like 4	1,69	381113
Trip11	thyroid hormone receptor interactor 11	1,69	109181
Maged2	melanoma antigen, family D, 2	1,69	80884
Pknox1	Pbx/knotted 1 homeobox	-1,69	18771
Ctsb	cathepsin B	1,69	13030
Nme7	non-metastatic cells 7, protein expressed in (nucleoside-diphosphate kinase)	1,69	171567
Rdh12	retinol dehydrogenase 12	1,69	77974
Hmgxb4	HMG box domain containing 4	-1,69	70823
Fgl1	fibrinogen-like protein 1	1,69	234199

Lemd3	LEM domain containing 3	1,69	380664
Pcbp4	poly(rC) binding protein 4	-1,69	59092
AW112010	expressed sequence AW112010	1,69	107350
Srl	sarcalumenin	-1,69	106393
Zfp592	zinc finger protein 592	-1,69	233410
Snx16	sorting nexin 16	1,69	74718
Dnajb6	DnaJ (Hsp40) homolog, subfamily B, member 6	1,69	23950
Tcn2	transcobalamin 2	1,69	21452
Gga3	golgi associated, gamma adaptin ear containing, ARF binding protein 3	-1,68	260302
Cspp1	centrosome and spindle pole associated protein 1	-1,68	211660
Eif4ebp1	eukaryotic translation initiation factor 4E binding protein 1	-1,68	13685
Ccdc93	coiled-coil domain containing 93	1,68	70829
BC004004	cDNA sequence BC004004	-1,68	80748
1700021F05Rik	RIKEN cDNA 1700021F05 gene	-1,68	67851
Gpr172b	G protein-coupled receptor 172B	-1,68	52710
Elovl1	elongation of very long chain fatty acids (FEN1/Elo2, SUR4/Elo3, yeast)-like 1	-1,68	54325
Aldh18a1	aldehyde dehydrogenase 18 family, member A1	-1,68	56454
Itga2b	integrin alpha 2b	1,68	16399
Fam100b	family with sequence similarity 100, member B	1,68	319370
Ikbke	inhibitor of kappaB kinase epsilon	1,68	56489
Yipf2	Yip1 domain family, member 2	1,68	74766
Lgals8	lectin, galactose binding, soluble 8	1,68	56048
Lrrc57	leucine rich repeat containing 57	1,68	66606
Ufd1l	ubiquitin fusion degradation 1 like	1,68	22230
Fbp1	fructose bisphosphatase 1	1,68	14121
Zfp202	zinc finger protein 202	-1,68	80902
Fam118a	family with sequence similarity 118, member A	-1,68	73225
Hist3h2a	histone cluster 3, H2a	1,68	319162
Tmem18	transmembrane protein 18	1,68	211986
Tmem68	transmembrane protein 68	1,68	72098
Kctd6	potassium channel tetramerisation domain containing 6	1,68	71393
Phc2	polyhomeotic-like 2 (Drosophila)	-1,68	54383
Degs1	degenerative spermatocyte homolog 1 (Drosophila)	1,67	13244
Mbd4	methyl-CpG binding domain protein 4	1,67	17193
Lig3	ligase III, DNA, ATP-dependent	-1,67	16882
Cast	calpastatin	1,67	12380
1700029I01Rik	RIKEN cDNA 1700029I01 gene	-1,67	70005
Dhrs4	dehydrogenase/reductase (SDR family) member 4	1,67	28200
Mobkl2c	MOB1, Mps One Binder kinase activator-like 2C (yeast)	1,67	100465
Nsun6	NOL1/NOP2/Sun domain family member 6	1,67	74455
Tecpr2	tectonin beta-propeller repeat containing 2	-1,67	104859
Nudt2	nudix (nucleoside diphosphate linked moiety X)-type motif 2	-1,67	66401
Herpud1	homocysteine-inducible, endoplasmic reticulum stress-inducible, ubiquitin-like domain member 1	1,67	64209
D4Wsu53e	DNA segment, Chr 4, Wayne State University 53, expressed	1,67	27981
Arrb2	arrestin, beta 2	-1,67	216869
2010305A19Rik	RIKEN cDNA 2010305A19 gene	-1,67	69893

Mylpf	myosin light chain, phosphorylatable, fast skeletal muscle	-1,67	17907
Mllt10	myeloid/lymphoid or mixed-lineage leukemia (trithorax homolog, Drosophila); translocated to, 10	-1,67	17354
Angel2	angel homolog 2 (Drosophila)	-1,67	52477
Nedd4l	neural precursor cell expressed, developmentally down-regulated gene 4-like	1,67	83814
Rab27a	RAB27A, member RAS oncogene family	1,67	11891
Tmem41a	transmembrane protein 41a	-1,67	66664
Trim37	tripartite motif-containing 37	-1,67	68729
Ctso	cathepsin O	1,67	229445
Fkbp3	FK506 binding protein 3	-1,67	30795
Trim12c	tripartite motif-containing 12C	1,67	319236
Git1	G protein-coupled receptor kinase-interactor 1	-1,67	216963
Mblac2	metallo-beta-lactamase domain containing 2	-1,67	72852
Armc10	armadillo repeat containing 10	-1,66	67211
Lpin1	lipin 1	1,66	14245
Rgl1	ral guanine nucleotide dissociation stimulator,-like 1	1,66	19731
Gm13845	predicted gene 13845	1,66	########
Slc7a1	solute carrier family 7 (cationic amino acid transporter, y+ system), member 1	-1,66	11987
BC017643	cDNA sequence BC017643	1,66	217370
Fam136a	family with sequence similarity 136, member A	-1,66	66488
Kpnb1	karyopherin (importin) beta 1	-1,66	16211
Tnfrsf22	tumor necrosis factor receptor superfamily, member 22	1,66	79202
Zfp712	zinc finger protein 712	-1,66	78251
Stxbp4	syntaxin binding protein 4	1,66	20913
Gpatch8	G patch domain containing 8	-1,66	237943
4930428B01Rik	RIKEN cDNA 4930428B01 gene	1,66	73854
Grap	GRB2-related adaptor protein	1,66	71520
Tmed4	transmembrane emp24 protein transport domain containing 4	1,66	103694
Nlrc5	NLR family, CARD domain containing 5	1,66	434341
A130064L14Rik	RIKEN cDNA A130064L14 gene	-1,66	320257
Mcart1	mitochondrial carrier triple repeat 1	1,65	230125
Ppm1a	protein phosphatase 1A, magnesium dependent, alpha isoform	-1,65	19042
Chmp1b	chromatin modifying protein 1B	1,65	67064
2310001H17Rik	RIKEN cDNA 2310001H17 gene	-1,65	76432
Sip1	survival of motor neuron protein interacting protein 1	1,65	66603
Tax1bp3	Tax1 (human T-cell leukemia virus type I) binding protein 3	1,65	76281
Tmem128	transmembrane protein 128	1,65	66309
Zfp316	zinc finger protein 316	-1,65	54201
C8g	complement component 8, gamma polypeptide	1,65	69379
Icam1	intercellular adhesion molecule 1	1,65	15894
3110043O21Rik	RIKEN cDNA 3110043O21 gene	1,65	73205
Heatr5a	HEAT repeat containing 5A	-1,65	320487
Gm166	predicted gene 166	1,65	233899
Zfyve26	zinc finger, FYVE domain containing 26	1,65	211978
Casd1	CAS1 domain containing 1	-1,65	213819
Gba2	glucosidase beta 2	1,65	230101
Atxn7	ataxin 7	1,65	246103

Zfp282	zinc finger protein 282	-1,65	101095
H2afv	H2A histone family, member V	1,65	77605
Dgcr8	DiGeorge syndrome critical region gene 8	-1,65	94223
Usp42	ubiquitin specific peptidase 42	-1,65	76800
Prkd2	protein kinase D2	-1,65	101540
Tpk1	thiamine pyrophosphokinase	-1,64	29807
Hmg20a	high mobility group 20A	-1,64	66867
Yipf6	Yip1 domain family, member 6	1,64	77929
Unc13b	unc-13 homolog B (C. elegans)	1,64	22249
Nutf2	nuclear transport factor 2	-1,64	68051
Smg6	Smg-6 homolog, nonsense mediated mRNA decay factor (C. elegans)	-1,64	103677
Rnf138	ring finger protein 138	-1,64	56515
Sdr42e1	short chain dehydrogenase/reductase family 42E, member 1	1,64	74032
Ogfrl1	opioid growth factor receptor-like 1	-1,64	70155
Phf20	PHD finger protein 20	-1,64	228829
Hip1	huntingtin interacting protein 1	-1,64	215114
Prkar2b	protein kinase, cAMP dependent regulatory, type II beta	-1,64	19088
Ppm1k	protein phosphatase 1K (PP2C domain containing)	1,64	243382
Med12l	mediator of RNA polymerase II transcription, subunit 12 homolog (yeast)-like	1,64	329650
Dera	2-deoxyribose-5-phosphate aldolase homolog (C. elegans)	-1,64	232449
Commd7	COMM domain containing 7	-1,64	99311
Gtdc1	glycosyltransferase-like domain containing 1	-1,64	227835
Cyp2c44	cytochrome P450, family 2, subfamily c, polypeptide 44	1,64	226143
Nup188	nucleoporin 188	-1,64	227699
Naip2	NLR family, apoptosis inhibitory protein 2	1,64	17948
Memo1	mediator of cell motility 1	-1,64	76890
Ctbp2	C-terminal binding protein 2	-1,64	13017
Mgat5	mannoside acetylglucosaminyltransferase 5	1,64	107895
Vegfb	vascular endothelial growth factor B	-1,64	22340
Tgfbr3	transforming growth factor, beta receptor III	-1,64	21814
Sp100	nuclear antigen Sp100	1,64	20684
Wbp4	WW domain binding protein 4	-1,64	22380
Tnfaip2	tumor necrosis factor, alpha-induced protein 2	1,64	21928
Nup62	nucleoporin 62	-1,64	18226
Spty2d1	SPT2, Suppressor of Ty, domain containing 1 (S. cerevisiae)	1,64	101685
A430103D13Rik	RIKEN cDNA A430103D13 gene	-1,64	77775
Fnbp4	formin binding protein 4	1,63	55935
Mtf1	metal response element binding transcription factor 1	1,63	17764
Akr1b3	aldo-keto reductase family 1, member B3 (aldose reductase)	1,63	11677
Ncdn	neurochondrin	-1,63	26562
Slc41a2	solute carrier family 41, member 2	1,63	338365
Icam4	intercellular adhesion molecule 4, Landsteiner-Wiener blood group	-1,63	78369
Rbmx2	RNA binding motif protein, X-linked 2	-1,63	209003
Ccdc97	coiled-coil domain containing 97	1,63	52132
4930592C13Rik	RIKEN cDNA 4930592C13 gene	1,63	381498

Naip5	NLR family, apoptosis inhibitory protein 5	1,63	17951
Cyfip1	cytoplasmic FMR1 interacting protein 1	-1,63	20430
4632428N05Rik	RIKEN cDNA 4632428N05 gene	1,63	74048
BC062258	cDNA sequence BC062258	-1,63	408063
Podxl	podocalyxin-like	-1,63	27205
Xiap	X-linked inhibitor of apoptosis	-1,63	11798
Mettl7a1	methyltransferase like 7A1	1,63	70152
Heatr1	HEAT repeat containing 1	-1,63	217995
Irf3	interferon regulatory factor 3	1,63	54131
Exd1	exonuclease 3'-5' domain containing 1	1,63	241624
Abhd8	abhydrolase domain containing 8	-1,63	64296
Scd2	stearoyl-Coenzyme A desaturase 2	-1,63	20250
Egr3	early growth response 3	-1,63	13655
Abhd14a	abhydrolase domain containing 14A	-1,63	68644
Nr4a2	nuclear receptor subfamily 4, group A, member 2	1,63	18227
Sema4d	sema domain, immunoglobulin domain (Ig), transmembrane domain (TM) and short cytoplasmic domain, (semaphorin) 4D	-1,63	20354
Mios	missing oocyte, meiosis regulator, homolog (Drosophila)	-1,63	252875
Tsga14	testis specific gene A14	-1,63	83922
Pdzd2	PDZ domain containing 2	1,63	68070
BB236558	expressed sequence BB236558	1,62	102836
Gba	glucosidase, beta, acid	1,62	14466
Cpeb2	cytoplasmic polyadenylation element binding protein 2	1,62	231207
Intu	inturned planar cell polarity effector homolog (Drosophila)	1,62	380614
Yipf5	Yip1 domain family, member 5	1,62	67180
4933439F18Rik	RIKEN cDNA 4933439F18 gene	-1,62	66771
Bri3	brain protein I3	-1,62	55950
Glipr2	GLI pathogenesis-related 2	1,62	384009
Ap2a2	adaptor protein complex AP-2, alpha 2 subunit	1,62	11772
Csk	c-src tyrosine kinase	-1,62	12988
Churc1	churchill domain containing 1	1,62	211151
Fam50a	family with sequence similarity 50, member A	1,62	108160
Col23a1	collagen, type XXIII, alpha 1	1,62	237759
Sec11c	SEC11 homolog C (S. cerevisiae)	1,61	66286
Reep3	receptor accessory protein 3	1,61	28193
9130008F23Rik	RIKEN cDNA 9130008F23 gene	1,61	71583
Ola1	Obg-like ATPase 1	-1,61	67059
Mark4	MAP/microtubule affinity-regulating kinase 4	-1,61	232944
Zeb2	zinc finger E-box binding homeobox 2	-1,61	24136
Lage3	L antigen family, member 3	-1,61	66192
Fnip2	folliculin interacting protein 2	-1,61	329679
Brd8	bromodomain containing 8	1,61	78656
Rap1b	RAS related protein 1b	-1,61	215449
Erap1	endoplasmic reticulum aminopeptidase 1	-1,61	80898
Cml1	camello-like 1	1,61	66116
Capns1	calpain, small subunit 1	1,61	12336
Pgap2	post-GPI attachment to proteins 2	-1,61	233575
Fbxo3	F-box protein 3	-1,61	57443
Gt(ROSA)26Sor	gene trap ROSA 26, Philippe Soriano	-1,61	14910

Trmt2a	TRM2 tRNA methyltransferase 2 homolog A (S. cerevisiae)	1,61	15547
Slc25a39	solute carrier family 25, member 39	-1,61	68066
Sell	selectin, lymphocyte	-1,60	20343
Tpra1	transmembrane protein, adipocyte asscociated 1	1,60	24100
4930528A17Rik	RIKEN cDNA 4930528A17 gene	1,60	67735
Cebpb	CCAAT/enhancer binding protein (C/EBP), beta	1,60	12608
Ftsjd2	FtsJ methyltransferase domain containing 2	-1,60	74157
Acn9	ACN9 homolog (S. cerevisiae)	-1,60	71238
Cul3	cullin 3	-1,60	26554
Tmem202	transmembrane protein 202	1,60	73893
Nudt12	nudix (nucleoside diphosphate linked moiety X)-type motif 12	1,60	67993
Nbeal2	neurobeachin-like 2	-1,60	235627
Gipc1	GIPC PDZ domain containing family, member 1	-1,60	67903
1500031L02Rik	RIKEN cDNA 1500031L02 gene	1,60	66994
Mtmr14	myotubularin related protein 14	1,60	97287
Zfpm1	zinc finger protein, multitype 1	-1,60	22761
Ubqln4	ubiquilin 4	-1,60	94232
8430427H17Rik	RIKEN cDNA 8430427H17 gene	-1,60	329540
Thra	thyroid hormone receptor alpha	-1,60	21833
1700010I14Rik	RIKEN cDNA 1700010I14 gene	1,60	66931
Pramef8	PRAME family member 8	1,60	242736
Ccdc120	coiled-coil domain containing 120	1,60	54648
Nostrin	nitric oxide synthase trafficker	1,60	329416
Tm6sf1	transmembrane 6 superfamily member 1	1,60	107769
3830406C13Rik	RIKEN cDNA 3830406C13 gene	-1,60	218734
Fam120a	family with sequence similarity 120, member A	-1,60	218236
Dpcd	deleted in primary ciliary dyskinesia	-1,60	226162
Secisbp2l	SECIS binding protein 2-like	1,60	70354
Rfesd	Rieske (Fe-S) domain containing	1,60	218341
Atp6v0a1	ATPase, H+ transporting, lysosomal V0 subunit A1	1,60	11975
Qrsl1	glutaminyl-tRNA synthase (glutamine-hydrolyzing)-like 1	-1,60	76563
Osbpl11	oxysterol binding protein-like 11	1,60	106326
Gyk	glycerol kinase	-1,60	14933
Naif1	nuclear apoptosis inducing factor 1	-1,60	71254
Scnm1	sodium channel modifier 1	1,60	69269
Mycn	v-myc myelocytomatosis viral related oncogene, neuroblastoma derived (avian)	-1,59	18109
Rnd1	Rho family GTPase 1	1,59	223881
Gadd45b	growth arrest and DNA-damage-inducible 45 beta	1,59	17873
Zfp687	zinc finger protein 687	-1,59	78266
Ltb4r2	leukotriene B4 receptor 2	1,59	57260
Rap1gds1	RAP1, GTP-GDP dissociation stimulator 1	-1,59	229877
Itgb1	integrin beta 1 (fibronectin receptor beta)	-1,59	16412
Zfp40	zinc finger protein 40	-1,59	22700
BC027231	cDNA sequence BC027231	-1,59	212547
Hivep2	human immunodeficiency virus type I enhancer binding protein 2	1,59	15273
Abcb6	ATP-binding cassette, sub-family B (MDR/TAP), member 6	1,59	74104
Creb3l2	cAMP responsive element binding protein 3-like 2	1,59	208647

Ppp1r16b	protein phosphatase 1, regulatory (inhibitor) subunit 16B	1,59	228852
Akap13	A kinase (PRKA) anchor protein 13	-1,59	75547
Slc35a5	solute carrier family 35, member A5	-1,59	74102
Cfl2	cofilin 2, muscle	1,59	12632
Bid	BH3 interacting domain death agonist	-1,59	12122
Trim68	tripartite motif-containing 68	-1,59	101700
Sirt7	sirtuin 7 (silent mating type information regulation 2, homolog) 7 (S. cerevisiae)	1,59	209011
Nqo2	NAD(P)H dehydrogenase, quinone 2	1,59	18105
Nipsnap1	4-nitrophenylphosphatase domain and non-neuronal SNAP25-like protein homolog 1 (C. elegans)	-1,59	18082
Fam82b	family with sequence similarity 82, member B	1,59	66302
Mtap7	microtubule-associated protein 7	1,59	17761
Hace1	HECT domain and ankyrin repeat containing, E3 ubiquitin protein ligase 1	1,59	209462
Ccdc124	coiled-coil domain containing 124	-1,59	234388
Cnpy2	canopy 2 homolog (zebrafish)	1,58	56530
Hrc	histidine rich calcium binding protein	1,58	15464
Inpp5k	inositol polyphosphate 5-phosphatase K	1,58	19062
Arap3	ArfGAP with RhoGAP domain, ankyrin repeat and PH domain 3	-1,58	106952
2700049A03Rik	RIKEN cDNA 2700049A03 gene	1,58	76967
Nek3	NIMA (never in mitosis gene a)-related expressed kinase 3	-1,58	23954
Stk3	serine/threonine kinase 3 (Ste20, yeast homolog)	-1,58	56274
C2cd2l	C2 calcium-dependent domain containing 2-like	1,58	71764
Alg11	asparagine-linked glycosylation 11 homolog (yeast, alpha-1,2-mannosyltransferase)	-1,58	207958
Asxl1	additional sex combs like 1 (Drosophila)	-1,58	228790
Myc	myelocytomatosis oncogene	-1,58	17869
Kdm6a	lysine (K)-specific demethylase 6A	1,58	22289
Spns2	spinster homolog 2 (Drosophila)	-1,58	216892
Wwp2	WW domain containing E3 ubiquitin protein ligase 2	-1,58	66894
Nfkbiz	nuclear factor of kappa light polypeptide gene enhancer in B-cells inhibitor, zeta	1,58	80859
Chic2	cysteine-rich hydrophobic domain 2	1,58	74277
Kat5	K(lysine) acetyltransferase 5	-1,58	81601
2410022L05Rik	RIKEN cDNA 2410022L05 gene	1,58	66423
Pcm1	pericentriolar material 1	-1,58	18536
Esrra	estrogen related receptor, alpha	-1,58	26379
Rasal3	RAS protein activator like 3	1,58	320484
Lyz2	lysozyme 2	1,58	17105
S100a6	S100 calcium binding protein A6 (calcyclin)	1,58	20200
Ascc3	activating signal cointegrator 1 complex subunit 3	-1,58	77987
L2hgdh	L-2-hydroxyglutarate dehydrogenase	-1,58	217666
Slc48a1	solute carrier family 48 (heme transporter), member 1	-1,58	67739
Pfkm	phosphofructokinase, muscle	-1,58	18642
Mettl1	methyltransferase like 1	-1,58	17299
Ikbkg	inhibitor of kappaB kinase gamma	1,58	16151
Idh3a	isocitrate dehydrogenase 3 (NAD+) alpha	-1,58	67834
Zfp69	zinc finger protein 69	1,58	381549

Atl3	atlastin GTPase 3	-1,58	109168
Gimap6	GTPase, IMAP family member 6	1,58	231931
1110012D08Rik	RIKEN cDNA 1110012D08 gene	1,58	73827
Pcmtd1	protein-L-isoaspartate (D-aspartate) O-methyltransferase domain containing 1	1,58	319263
Klf16	Kruppel-like factor 16	-1,57	118445
Coq3	coenzyme Q3 homolog, methyltransferase (yeast)	-1,57	230027
Snrnp27	small nuclear ribonucleoprotein 27 (U4/U6.U5)	-1,57	66618
Irak3	interleukin-1 receptor-associated kinase 3	1,57	73914
Cenpt	centromere protein T	1,57	320394
Tmub2	transmembrane and ubiquitin-like domain containing 2	1,57	72053
Zadh2	zinc binding alcohol dehydrogenase, domain containing 2	-1,57	225791
Ezh1	enhancer of zeste homolog 1 (Drosophila)	-1,57	14055
Rnf19b	ring finger protein 19B	1,57	75234
Svip	small VCP/p97-interacting protein	-1,57	75744
Srcrb4d	scavenger receptor cysteine rich domain containing, group B (4 domains)	1,57	109267
Khnyn	KH and NYN domain containing	-1,57	219094
Mblac1	metallo-beta-lactamase domain containing 1	-1,57	330216
Mpp5	membrane protein, palmitoylated 5 (MAGUK p55 subfamily member 5)	-1,57	56217
Tk2	thymidine kinase 2, mitochondrial	1,57	57813
Tubb6	tubulin, beta 6	1,57	67951
Bpifb1	BPI fold containing family B, member 1	1,57	228801
Ccdc134	coiled-coil domain containing 134	1,57	76457
6230400D17Rik	RIKEN cDNA 6230400D17 gene	1,57	76133
Ctsa	cathepsin A	1,57	19025
Furin	furin (paired basic amino acid cleaving enzyme)	1,57	18550
Pter	phosphotriesterase related	-1,57	19212
Rgs12	regulator of G-protein signaling 12	1,57	71729
Nat2	N-acetyltransferase 2 (arylamine N-acetyltransferase)	1,57	17961
Ctdsp1	CTD (carboxy-terminal domain, RNA polymerase II, polypeptide A) small phosphatase 1	-1,57	227292
Agxt2l2	alanine-glyoxylate aminotransferase 2-like 2	-1,57	72947
Ankrd9	ankyrin repeat domain 9	-1,57	74251
Fhit	fragile histidine triad gene	1,56	14198
Fam164a	family with sequence similarity 164, member A	-1,56	67306
Gnpda1	glucosamine-6-phosphate deaminase 1	1,56	26384
Tgfb1	transforming growth factor, beta 1	-1,56	21803
Eml5	echinoderm microtubule associated protein like 5	1,56	319670
Tollip	toll interacting protein	1,56	54473
Sec24d	Sec24 related gene family, member D (S. cerevisiae)	1,56	69608
Ppp1r14b	protein phosphatase 1, regulatory (inhibitor) subunit 14B	-1,56	18938
Dut	deoxyuridine triphosphatase	-1,56	110074
Tmem63a	transmembrane protein 63a	-1,56	208795
Tspan4	tetraspanin 4	-1,56	64540
Ccdc111	coiled-coil domain containing 111	-1,56	408022
A230046K03Rik	RIKEN cDNA A230046K03 gene	-1,56	319277
Dpf3	D4, zinc and double PHD fingers, family 3	1,56	70127
Abhd11	abhydrolase domain containing 11	-1,56	68758

Ltb4r1	leukotriene B4 receptor 1	1,56	16995
Sec24b	Sec24 related gene family, member B (S. cerevisiae)	-1,56	99683
Atad1	ATPase family, AAA domain containing 1	-1,56	67979
Tbc1d15	TBC1 domain family, member 15	1,56	66687
Siah1a	seven in absentia 1A	-1,56	20437
2900093K20Rik	RIKEN cDNA 2900093K20 gene	1,56	68037
Nr1h2	nuclear receptor subfamily 1, group H, member 2	1,56	22260
Manbal	mannosidase, beta A, lysosomal-like	-1,56	69161
Zfp707	zinc finger protein 707	1,56	69020
Lpcat1	lysophosphatidylcholine acyltransferase 1	-1,56	210992
Reep6	receptor accessory protein 6	-1,56	70335
Sqstm1	sequestosome 1	1,56	18412
Mpv17	MpV17 mitochondrial inner membrane protein	1,56	17527
Emilin2	elastin microfibril interfacer 2	-1,56	246707
Zbtb44	zinc finger and BTB domain containing 44	-1,56	235132
Sla	src-like adaptor	1,56	20491
Mre11a	meiotic recombination 11 homolog A (S. cerevisiae)	-1,56	17535
Insig2	insulin induced gene 2	1,56	72999
Arsk	arylsulfatase K	1,56	77041
Wdr37	WD repeat domain 37	1,56	207615
Ostm1	osteopetrosis associated transmembrane protein 1	1,56	14628
Phf7	PHD finger protein 7	1,56	71838
Endod1	endonuclease domain containing 1	-1,56	71946
Exoc3l	exocyst complex component 3-like	1,56	277978
Plch1	phospholipase C, eta 1	1,55	269437
Galnt2	UDP-N-acetyl-alpha-D-galactosamine:polypeptide N-acetylgalactosaminyltransferase 2	-1,55	108148
Mavs	mitochondrial antiviral signaling protein	1,55	228607
Zfp768	zinc finger protein 768	-1,55	233890
Nufip2	nuclear fragile X mental retardation protein interacting protein 2	-1,55	68564
Abca4	ATP-binding cassette, sub-family A (ABC1), member 4	1,55	11304
March2	membrane-associated ring finger (C3HC4) 2	1,55	224703
Dhodh	dihydroorotate dehydrogenase	-1,55	56749
Hbegf	heparin-binding EGF-like growth factor	1,55	15200
Thap2	THAP domain containing, apoptosis associated protein 2	-1,55	66816
Zscan29	zinc finger SCAN domains 29	1,55	99334
Spryd3	SPRY domain containing 3	-1,55	223918
Mgst2	microsomal glutathione S-transferase 2	1,55	211666
2210021J22Rik	RIKEN cDNA 2210021J22 gene	-1,55	72355
Chmp1a	chromatin modifying protein 1A	-1,55	234852
Fam63b	family with sequence similarity 63, member B	1,55	235461
Srek1ip1	splicing regulatory glutamine/lysine-rich protein 1interacting protein 1	1,55	67288
Sfi1	Sfi1 homolog, spindle assembly associated (yeast)	-1,55	78887
Ubr4	ubiquitin protein ligase E3 component n-recognin 4	-1,55	69116
Oas2	2'-5' oligoadenylate synthetase 2	1,55	246728
Arl13b	ADP-ribosylation factor-like 13B	-1,55	68146
Strada	STE20-related kinase adaptor alpha	-1,55	72149
Lmo2	LIM domain only 2	1,55	16909
Fkbp14	FK506 binding protein 14	1,55	231997

Ppp2r4	protein phosphatase 2A, regulatory subunit B (PR 53)	-1,55	110854
Gigyf2	GRB10 interacting GYF protein 2	1,55	227331
Irgq	immunity-related GTPase family, Q	1,55	210146
Arhgap9	Rho GTPase activating protein 9	1,55	216445
Ccdc104	coiled-coil domain containing 104	1,55	216618
Lphn1	latrophilin 1	-1,55	330814
Abhd4	abhydrolase domain containing 4	1,55	105501
Mina	myc induced nuclear antigen	-1,55	67014
Rab3d	RAB3D, member RAS oncogene family	-1,55	19340
4933411E08Rik	RIKEN cDNA 4933411E08 gene	1,55	71067
Alms1	Alstrom syndrome 1 homolog (human)	-1,55	236266
2810422J05Rik	RIKEN cDNA 2810422J05 gene	-1,55	75620
Klhl8	kelch-like 8 (Drosophila)	1,55	246293
Pi4k2b	phosphatidylinositol 4-kinase type 2 beta	1,55	67073
Tubd1	tubulin, delta 1	1,55	56427
Mfng	MFNG O-fucosylpeptide 3-beta-N-acetylglucosaminyltransferase	-1,55	17305
Ddb2	damage specific DNA binding protein 2	1,54	107986
Irak1bp1	interleukin-1 receptor-associated kinase 1 binding protein 1	1,54	65099
Zfand2a	zinc finger, AN1-type domain 2A	1,54	100494
Sh2d5	SH2 domain containing 5	-1,54	230863
Dhrs3	dehydrogenase/reductase (SDR family) member 3	1,54	20148
Tmem164	transmembrane protein 164	-1,54	209497
Wdr47	WD repeat domain 47	-1,54	99512
Hint3	histidine triad nucleotide binding protein 3	1,54	66847
Cd55	CD55 antigen	-1,54	13136
Tmem106b	transmembrane protein 106B	1,54	71900
Uevld	UEV and lactate/malate dehyrogenase domains	-1,54	54122
Cd68	CD68 antigen	1,54	12514
1700080G18Rik	RIKEN cDNA 1700080G18 gene	1,54	73533
Dcakd	dephospho-CoA kinase domain containing	-1,54	68087
1110029L17Rik	RIKEN cDNA 1110029L17 gene	-1,54	68722
Osgepl1	O-sialoglycoprotein endopeptidase-like 1	1,54	72085
Zfp874b	zinc finger protein 874b	1,54	408067
Tlr4	toll-like receptor 4	1,54	21898
Ikzf2	IKAROS family zinc finger 2	1,54	22779
Dctpp1	dCTP pyrophosphatase 1	-1,54	66422
Muted	muted	-1,54	17828
Maz	MYC-associated zinc finger protein (purine-binding transcription factor)	-1,54	17188
Rbmxrt	RNA binding motif protein, X chromosome retrogene	-1,54	19656
Trim30a	tripartite motif-containing 30A	1,54	20128
Klhl5	kelch-like 5 (Drosophila)	-1,54	71778
Slc35d1	solute carrier family 35 (UDP-glucuronic acid/UDP-N-acetylgalactosamine dual transporter), member D1	1,54	242585
Ergic1	endoplasmic reticulum-golgi intermediate compartment (ERGIC) 1	-1,54	67458
Pnpla6	patatin-like phospholipase domain containing 6	1,54	50767
Baat1	BRCA1-associated ATM activator 1	-1,54	231841
Ift80	intraflagellar transport 80 homolog (Chlamydomonas)	1,54	68259

Zfp229	zinc finger protein	1,54	381067
Wdr91	WD repeat domain 91	-1,54	101240
Terf2ip	telomeric repeat binding factor 2, interacting protein	1,54	57321
Clk1	CDC-like kinase 1	1,54	12747
Mga	MAX gene associated	-1,54	29808
Asb1	ankyrin repeat and SOCS box-containing 1	-1,54	65247
Clpx	caseinolytic peptidase X (E.coli)	1,54	270166
Dus4l	dihydrouridine synthase 4-like (S. cerevisiae)	-1,54	71916
Pkig	protein kinase inhibitor, gamma	1,53	18769
Tpst2	protein-tyrosine sulfotransferase 2	-1,53	22022
Xrcc6bp1	XRCC6 binding protein 1	-1,53	68876
Cad	carbamoyl-phosphate synthetase 2, aspartate transcarbamylase, and dihydroorotase	-1,53	69719
Creg1	cellular repressor of E1A-stimulated genes 1	1,53	433375
Hddc2	HD domain containing 2	-1,53	69692
2610020C07Rik	RIKEN cDNA 2610020C07 gene	1,53	69918
Casp9	caspase 9	-1,53	12371
Homez	homeodomain leucine zipper-encoding gene	-1,53	239099
Ap1s1	adaptor protein complex AP-1, sigma 1	-1,53	11769
Dgat1	diacylglycerol O-acyltransferase 1	1,53	13350
Adcy7	adenylate cyclase 7	1,53	11513
Necap2	NECAP endocytosis associated 2	1,53	66147
Cd99l2	CD99 antigen-like 2	-1,53	171486
Asb3	ankyrin repeat and SOCS box-containing 3	1,53	65257
Insl6	insulin-like 6	1,53	27356
Mlf2	myeloid leukemia factor 2	-1,53	30853
Msl1	male-specific lethal 1 homolog (Drosophila)	-1,53	74026
Fam36a	family with sequence similarity 36, member A	-1,53	66359
Srgn	serglycin	1,53	19073
Zdhhc20	zinc finger, DHHC domain containing 20	1,53	75965
Pex16	peroxisomal biogenesis factor 16	1,53	18633
Otud7b	OTU domain containing 7B	-1,53	229603
Kifap3	kinesin-associated protein 3	-1,53	16579
Pkd2l2	polycystic kidney disease 2-like 2	1,53	53871
Txn2	thioredoxin 2	-1,53	56551
Cr1l	complement component (3b/4b) receptor 1-like	1,53	12946
Rnf185	ring finger protein 185	1,53	193670
Epb4.1l2	erythrocyte protein band 4.1-like 2	-1,53	13822
Cand1	cullin associated and neddylation disassociated 1	-1,53	71902
Tmem106c	transmembrane protein 106C	-1,53	380967
Gtf2a1	general transcription factor II A, 1	-1,53	83602
Bet1	blocked early in transport 1 homolog (S. cerevisiae)	1,53	12068
Cln6	ceroid-lipofuscinosis, neuronal 6	-1,53	76524
Rbm18	RNA binding motif protein 18	1,53	67889
Id1	inhibitor of DNA binding 1	-1,53	15901
Atp10d	ATPase, class V, type 10D	-1,53	231287
1700123O20Rik	RIKEN cDNA 1700123O20 gene	1,53	58248
Ankrd11	ankyrin repeat domain 11	-1,53	77087
Sat1	spermidine/spermine N1-acetyl transferase 1	1,53	20229
Ptprj	protein tyrosine phosphatase, receptor type, J	-1,52	19271
Chd2	chromodomain helicase DNA binding protein 2	1,52	244059
Matr3	matrin 3	-1,52	17184
Sap30l	SAP30-like	-1,52	50724

Gbas	glioblastoma amplified sequence	-1,52	14467
Pla2g12a	phospholipase A2, group XIIA	-1,52	66350
Ttc39a	tetratricopeptide repeat domain 39A	1,52	230603
9530053H05Rik	RIKEN cDNA 9530053H05 gene	1,52	78425
Atg14	VATG14 autophagy related 14 homolog (S. cerevisiae)	1,52	#########
Tnfrsf13b	tumor necrosis factor receptor superfamily, member 13b	1,52	57916
Chchd4	coiled-coil-helix-coiled-coil-helix domain containing 4	-1,52	72170
1810014B01Rik	RIKEN cDNA 1810014B01 gene	-1,52	66263
Nomo1	nodal modulator 1	-1,52	211548
Csf2	colony stimulating factor 2 (granulocyte-macrophage)	1,52	12981
Xaf1	XIAP associated factor 1	1,52	327959
Mbtps2	membrane-bound transcription factor peptidase, site 2	-1,52	270669
Slc13a2	solute carrier family 13 (sodium-dependent dicarboxylate transporter), member 2	1,52	20500
Lrrc61	leucine rich repeat containing 61	-1,52	243371
Ranbp3	RAN binding protein 3	-1,52	71810
Zfp830	zinc finger protein 830	1,52	66983
5430439M09Rik	RIKEN cDNA 5430439M09 gene	-1,52	71348
Inpp5e	inositol polyphosphate-5-phosphatase E	-1,52	64436
Sel1l	sel-1 suppressor of lin-12-like (C. elegans)	1,52	20338
Gstt4	glutathione S-transferase, theta 4	1,52	75886
Zbtb34	zinc finger and BTB domain containing 34	-1,52	241311
Bfar	bifunctional apoptosis regulator	1,52	67118
Ttf1	transcription termination factor, RNA polymerase I	1,52	22130
Morn2	MORN repeat containing 2	1,52	378462
Polr3c	polymerase (RNA) III (DNA directed) polypeptide C	1,52	74414
Glcci1	glucocorticoid induced transcript 1	1,52	170772
Btbd10	BTB (POZ) domain containing 10	1,52	68815
Dennd5a	DENN/MADD domain containing 5A	1,52	19347
Sergef	secretion regulating guanine nucleotide exchange factor	-1,52	27414
Asb13	ankyrin repeat and SOCS box-containing 13	1,52	142688
Sap30	sin3 associated polypeptide	1,52	60406
Mta1	metastasis associated 1	-1,52	116870
Il2rg	interleukin 2 receptor, gamma chain	1,52	16186
Obfc2a	oligonucleotide/oligosaccharide-binding fold containing 2A	1,52	109019
Orai2	ORAI calcium release-activated calcium modulator 2	-1,52	269717
Casp2	caspase 2	-1,52	12366
Anxa1	annexin A1	1,52	16952
Megf8	multiple EGF-like-domains 8	-1,51	269878
Serf1	small EDRK-rich factor 1	-1,51	20365
Ubap1	ubiquitin-associated protein 1	-1,51	67123
E130306D19Rik	RIKEN cDNA E130306D19 gene	1,51	230098
Ethe1	ethylmalonic encephalopathy 1	1,51	66071
Sepn1	selenoprotein N, 1	1,51	74777
Wdr34	WD repeat domain 34	-1,51	71820
Serpinb6a	serine (or cysteine) peptidase inhibitor, clade B, member 6a	1,51	20719

Zmat3	zinc finger matrin type 3	1,51	22401
Bend3	BEN domain containing 3	-1,51	331623
Herc3	hect domain and RLD 3	-1,51	73998
Grlf1	glucocorticoid receptor DNA binding factor 1	-1,51	232906
Sp3	trans-acting transcription factor 3	-1,51	20687
Ttc15	tetratricopeptide repeat domain 15	-1,51	217449
Triobp	TRIO and F-actin binding protein	-1,51	110253
2310008H04Rik	RIKEN cDNA 2310008H04 gene	-1,51	224008
Susd1	sushi domain containing 1	-1,51	634731
Arl6	ADP-ribosylation factor-like 6	1,51	56297
Fn3k	fructosamine 3 kinase	1,51	63828
Pycrl	pyrroline-5-carboxylate reductase-like	-1,51	66194
4933407H18Rik	RIKEN cDNA 4933407H18 gene	1,51	71101
Gngt2	guanine nucleotide binding protein (G protein), gamma transducing activity polypeptide 2	1,51	14710
Tes	testis derived transcript	1,51	21753
Phf2	PHD finger protein 2	1,51	18676
Phf15	PHD finger protein 15	-1,51	76901
Atp6v1b2	ATPase, H+ transporting, lysosomal V1 subunit B2	1,51	11966
Icmt	isoprenylcysteine carboxyl methyltransferase	1,51	57295
3110052M02Rik	RIKEN cDNA 3110052M02 gene	1,51	73229
Rb1cc1	RB1-inducible coiled-coil 1	1,51	12421
Senp3	SUMO/sentrin specific peptidase 3	-1,51	80886
Clk4	CDC like kinase 4	1,51	12750
Zbed4	zinc finger, BED domain containing 4	-1,51	223773
Mau2	MAU2 chromatid cohesion factor homolog (C. elegans)	1,51	74549
Farsb	phenylalanyl-tRNA synthetase, beta subunit	-1,51	23874
Afg3l2	AFG3(ATPase family gene 3)-like 2 (yeast)	-1,51	69597
Fam129b	family with sequence similarity 129, member B	1,51	227737
Tlr6	toll-like receptor 6	1,51	21899
Ifnar1	interferon (alpha and beta) receptor 1	-1,51	15975
4930579G22Rik	RIKEN cDNA 4930579G22 gene	-1,51	69034
4921515J06Rik	RIKEN cDNA 4921515J06 gene	1,51	66715
Zc4h2	zinc finger, C4H2 domain containing	1,51	245522
Tifa	TRAF-interacting protein with forkhead-associated domain	1,51	211550
Cisd3	CDGSH iron sulfur domain 3	-1,51	217149
Dnajc22	DnaJ (Hsp40) homolog, subfamily C, member 22	1,51	72778
Map3k1	mitogen-activated protein kinase kinase kinase 1	1,51	26401
Ift74	intraflagellar transport 74 homolog (Chlamydomonas)	1,51	67694
Trim32	tripartite motif-containing 32	-1,51	69807
Gemin5	gem (nuclear organelle) associated protein 5	-1,51	216766
Nenf	neuron derived neurotrophic factor	-1,50	66208
Elovl6	ELOVL family member 6, elongation of long chain fatty acids (yeast)	-1,50	170439
Krt10	keratin 10	1,50	16661
Zfyve21	zinc finger, FYVE domain containing 21	-1,50	68520
Zfp444	zinc finger protein 444	-1,50	72667
Inppl1	inositol polyphosphate phosphatase-like 1	1,50	16332
Zc3h12c	zinc finger CCCH type containing 12C	1,50	244871
1600020E01Rik	RIKEN cDNA 1600020E01 gene	1,50	72012
Prrc2a	proline-rich coiled-coil 2A	-1,50	53761

Efcab4a	EF-hand calcium binding domain 4A	1,50	213573
Abhd5	abhydrolase domain containing 5	1,50	67469
Tmem108	transmembrane protein 108	-1,50	81907
Zfp386	zinc finger protein 386 (Kruppel-like)	-1,50	56220
Srpk2	serine/arginine-rich protein specific kinase 2	1,50	20817
Cdc25c	cell division cycle 25 homolog C (S. pombe)	1,50	12532
Kif13b	kinesin family member 13B	1,50	16554
Zfp236	zinc finger protein 236	-1,50	329002
Ube2h	ubiquitin-conjugating enzyme E2H	1,50	22214
Ccm2	cerebral cavernous malformation 2 homolog (human)	-1,50	216527
Cdk6	cyclin-dependent kinase 6	-1,50	12571
Hnrnpd	heterogeneous nuclear ribonucleoprotein D	-1,50	11991
Zfp182	zinc finger protein 182	1,50	319535
Samsn1	SAM domain, SH3 domain and nuclear localization signals, 1	1,50	67742
