# Supplementary material for: Overexpression of the Transcription Factor Sp1 Activates the OAS-RNAse L-RIG-I Pathway
Source: PLoS One. 2015 Mar 4;10(3):e0118551. doi: 10.1371/journal.pone.0118551 (PMC4349862; doi:10.1371/journal.pone.0118551)
Supplement: S2 Table — The gene list enrichment analysis from the Gene Ontology of the SP1 specific signature was performed with g:Profiler. The moderate hierarchical filtering used here allows a compact representation of gene list enrichment results. Significantly enriched GO terms containing less than 5 genes were excluded. (PDF) [file pone.0118551.s002.pdf]

S2 Table. Functional analysis of Sp1 signature.

Term ID	Term name	p-value	Term size	Overlap size	Overlap genes
GO:0044238	Primary metabolic process	1.11e-17	5444	379	NEO1,UPP1,GBP2,ARIH1,SERPINF1,CD74,JUN,TMEM173,ST8SIA4,IRF7,FAM129A,LATS2,GALNT3,GBP3,RGS2,SLC11A1,BTG2,TGM2,PRICKLE1,DDC,CDKL2,RBMS1,MYL4,TSSK6,AURKC,USP2,TRP53INP2,PTGR1,MUC1,ARID5A,EGR2,GCH1,PARP14,MECP2,PADI3,DPPA2,SGPL1,ABCA1,FOS,SGK3,OASL2,SP1,JHDM1D,PCYT1B,GMEB1,TRAF1,SFMBT1,KLF6,DDX58,PJA2,C3,E2F2,DUSP1,CTSS,GNPTG,JDP2,CELA1,TRP53INP1,MALAT1,PDP1,RAMP1,NCF1,PPP1R15A,TUBB2A,PPP3CB,CASP4,TRIM9,MMP9,CARHSP1,FGF23,MORC3,CEBPD,CREM,RASIP1,CSRNP2,FUCA2,ISG20,MTAP,HMOX1,GPX1,GSTT1,DPP7,ZFP639,RNASEL,ZKSCAN4,SIN3A,HERC6,NPL,CDKN2C,ABAT,PUS7L,ERBB3,NLN,0610031J06RIK,AKR1B10,PRMT7,RBPMS,ZFP36,RFXAP,RCOR3,NAPSA,GADD45A,RAB34,UBR2,ABCB1B,RENBP,CBFA2T3,ZFP473,SERAC1,DUB1,TNNI3,TRPC2,PARP9,ST3GAL4,GLA,LGALS3,BCL9,FBXO4,FBXO6,CCPG1,RNF167,PLD3,ECM1,CCND2,ESR1,NFKBIE,TRF,CLN3,IKZF4,TAF13,MPG,2610002J02RIK,LSS,DNASE2A,L3MBTL3,TCEANC,AHI1,PDPK1,DUSP16,TPP1,CTNS,SP4,PIK3CA,DEPDC1A,SUV420H2,MEX3D,TRMT2B,RUNX2,ZFP280D,HSD17B11,PIF1,DCTD,PINK1,CENPK,ATF5,IGF2BP2,TRIP4,RHBDD1,ADNP2,GTF2B,ZFP748,PBRM1,HEXIM2,SPIB,PPIP5K1,B3GNT5,CHAF1B,GMPR2,MND1,USP32,PBX2,ADPRH,NDST2,HSPA4L,MACF1,RAB13,RBL1,MAP3K8,GDPD1,NFE2,ZFP667,ZMYM2,SENP8,SOCS6,MVP,NHLRC1,TRPM7,MXD1,ELAVL1,FLCN,CSAD,MDFIC,HMG20B,RAB37,CREB3L1,TUBB1,EDN3,IMPACT,DNAJB9,ITGB3BP,HGFAC,GNPDA2,TDP1,GTF2F1,NFIC,RPP40,SLCO4A1,GALK1,SH3GLB1,PPARGC1B,NPEPPS,HIST2H2BE,TUBA8,DUSP10,NR1D2,ARF6,ZXDC,PIP4K2B,CAMKK2,PMM1,GDA,USP34,NAGK,ZKSCAN14,TRIB3,LIPA,ADA,BBS4,SENP7,MUM1,GPD1L,AMZ2,CREB3,EBPL,ZFP771,SERPINA3G,BPGM,PRR13,POLR2H,BBS12,ATXN7L3,TLR2,IMMP1L,APLP2,WDSUB1,GATAD2B,NSMCE1,TAB2,PIK3IP1,CDKL4,PKNOX1,CTSB,NME7,RDH12,LEMD3,ZFP592,DNAJB6,GGA3,EIF4EBP1,ELOVL1,ALDH18A1,IKBKE,UFD1L,FBP1,ZFP202,HIST3H2A,TMEM18,DEGS1,MBD4,LIG3,CAST,DHRS4,HERPUD1,ARRB2,MLLT10,NEDD4L,TRIM37,CTSO,FKBP3,GIT1,LPIN1,NLRC5,PPM1A,TAX1BP3,ZFP316,C8G,ICAM1,ZFYVE26,GBA2,ATXN7,H2AFV,DGCR8,USP42,PRKD2,HMG20A,SMG6,SDR42E1,PHF20,HIP1,PRKAR2B,PPM1K,MED12L,DERA,COMMD7,CTBP2,MGAT5,VEGFB,TGFBR3,SP100,WBP4,NUP62,MTF1,AKR1B3,XIAP,IRF3,EXD1,SCD2,EGR3,NR4A2,SEMA4D,GBA,CPEB2,CSK,CHURC1,SEC11C,OLA1,MARK4,ZEB2,FNIP2,BRD8,RAP1B,ERAP1,CAPNS1,PGAP2,CEBPB,CUL3,NUDT12,GIPC1,ZFPM1,UBQLN4,THRA,NOSTRIN,QRSL1,GYK,SCNM1,MYCN,GADD45B,ZFP687,RAP1GDS1,ITGB1,HIVEP2,CREB3L2,AKAP13,TRIM68,SIRT7,HACE1,INPP5K,ARAP3,NEK3,STK3,ASXL1,MYC,WWP2,NFKBIZ,KAT5,ESRRA,RASAL3,ASCC3,L2HGDH,PFKM,METTL1,IKBKG,IDH3A,PCMTD1,KLF16,COQ3,SNRNP27,IRAK3,EZH1
GO:0044237	Cellular metabolic process	2.47e-15	5416	369	NEO1,UPP1,GBP2,ARIH1,CD74,JUN,TMEM173,ST8SIA4,IRF7,FAM129A,LATS2,GALNT3,GBP3,RGS2,SLC11A1,BTG2,TGM2,PRICKLE1,DDC,CDKL2,RBMS1,MYL4,TSSK6,AURKC,USP2,TRP53INP2,CKMT1,PTGR1,MUC1,ARID5A,EGR2,GCH1,PARP14,MECP2,PADI3,DPPA2,SGPL1,ABCA1,FOS,SGK3,OASL2,SP1,JHDM1D,PCYT1B,GMEB1,TRAF1,SCOC,SFMBT1,KLF6,DDX58,PJA2,C3,E2F2,DUSP1,LYZ1,GNPTG,SEPP1,JDP2,CELA1,TRP53INP1,MALAT1,PDP1,MAP1LC3B,RAMP1,NCF1,PPP1R15A,TUBB2A,PPP3CB,TRIM9,CARHSP1,FGF23,MORC3,CEBPD,CREM,RASIP1,ITSN1,CSRNP2,ISG20,DGKA,MTAP,HMOX1,GPX1,GSTT1,ZFP639,RNASEL,ZKSCAN4,SIN3A,HERC6,ULBP1,NPL,CDKN2C,ABAT,PUS7L,ERBB3,0610031J06RIK,AKR1B10,PRMT7,RBPMS,PPOX,ZFP36,RFXAP,RCOR3,GADD45A,RAB34,UBR2,ABCB1B,RENBP,CBFA2T3,ZFP473,SERAC1,DUB1,TNNI3,TRPC2,PARP9,ST3GAL4,ARSB,GLA,LGALS3,BCL9,ERO1LB,FBXO4,FBXO6,CCPG1,RNF167,ECM1,CCND2,ESR1,NFKBIE,TRF,CLN3,IKZF4,TAF13,MPG,2610002J02RIK,DNASE2A,IRGM1,L3MBTL3,TCEANC,AHI1,PDPK1,DUSP16,TPP1,CTNS,SP4,PIK3CA,DEPDC1A,ATG12,SUV420H2,MEX3D,TRMT2B,RUNX2,ZFP280D,HSD17B11,PIF1,DCTD,PINK1,CENPK,ATF5,IGF2BP2,TRIP4,TYROBP,RHBDD1,ADNP2,GTF2B,ZFP748,PBRM1,HEXIM2,SPIB,PPIP5K1,B3GNT5,CHAF1B,GMPR2,MND1,PBX2,ADPRH,NDST2,TAPBP,HSPA4L,MACF1,RAB13,RBL1,WIPI1,MAP3K8,9430023L20RIK,GDPD1,NFE2,ZFYVE1,ZFP667,ZMYM2,SOCS6,MVP,NHLRC1,TRPM7,NTPCR,MXD1,ELAVL1,FLCN,CSAD,MDFIC,HMG20B,RAB37,CREB3L1,TUBB1,NBR1,EDN3,IMPACT,DNAJB9,ITGB3BP,RAB31,TDP1,GTF2F1,NFIC,RPP40,SLCO4A1,GALK1,SH3GLB1,PPARGC1B,PFKFB3,HIST2H2BE,TUBA8,DUSP10,NR1D2,ARF6,ZXDC,PIP4K2B,CAMKK2,GDA,USP34,NAGK,ZKSCAN14,CYB561D2,TRIB3,LIPA,ADA,CHMP5,MUM1,GPD1L,CREB3,ZFP771,BPGM,PRR13,POLR2H,BBS12,ATXN7L3,C330018D20RIK,TLR2,IMMP1L,APLP2,WDSUB1,GATAD2B,NSMCE1,TAB2,PIK3IP1,CDKL4,PKNOX1,NME7,RDH12,LEMD3,ZFP592,DNAJB6,TCN2,EIF4EBP1,ELOVL1,ALDH18A1,IKBKE,UFD1L,FBP1,ZFP202,HIST3H2A,TMEM18,DEGS1,MBD4,LIG3,DHRS4,HERPUD1,ARRB2,MLLT10,NEDD4L,TRIM37,FKBP3,GIT1,LPIN1,NLRC5,PPM1A,TAX1BP3,ZFP316,ICAM1,ZFYVE26,GBA2,ATXN7,H2AFV,DGCR8,USP42,PRKD2,TPK1,HMG20A,SMG6,PHF20,HIP1,PRKAR2B,PPM1K,MED12L,DERA,COMMD7,CTBP2,MGAT5,VEGFB,TGFBR3,SP100,WBP4,NUP62,MTF1,AKR1B3,XIAP,IRF3,EXD1,SCD2,EGR3,NR4A2,SEMA4D,GBA,CPEB2,CSK,CHURC1,SEC11C,OLA1,MARK4,ZEB2,FNIP2,BRD8,RAP1B,PGAP2,SLC25A39,CEBPB,CUL3,NUDT12,GIPC1,MTMR14,ZFPM1,UBQLN4,THRA,NOSTRIN,QRSL1,GYK,SCNM1,MYCN,GADD45B,ZFP687,RAP1GDS1,ITGB1,HIVEP2,ABCB6,CREB3L2,AKAP13,TRIM68,SIRT7,HACE1,CNPY2,INPP5K,ARAP3,NEK3,STK3,ASXL1,MYC,WWP2,NFKBIZ,KAT5,ESRRA,RASAL3
GO:0019222	Regulation of metabolic process	6.63e-11	3828	268	NEO1,PF4,SERPINF1,CD74,JUN,TMEM173,IRF7,FAM129A,LATS2,GBP3,RGS2,SLC11A1,CNR2,BTG2,PRICKLE1,MYL4,USP2,TRP53INP2,MUC1,ARID5A,EGR2,GCH1,PARP14,MECP2,DPPA2,ABCA1,FOS,SP1,JHDM1D,SPINT1,GMEB1,TRAF1,SCOC,SFMBT1,KLF6,DDX58,C3,E2F2,DUSP1,JDP2,CELA1,TRP53INP1,MALAT1,RAMP1,PPP1R15A,PPP3CB,CARHSP1,FGF23,MORC3,CEBPD,CREM,RASIP1,ITSN1,CSRNP2,HMOX1,GPX1,ZFP639,RNASEL,ZKSCAN4,SIN3A,ULBP1,CDKN2C,ERBB3,0610031J06RIK,PRMT7,RBPMS,ZFP36,RFXAP,RCOR3,GADD45A,UBR2,RENBP,CBFA2T3,ZFP473,TNNI3,GLA,BCL9,FBXO4,CCPG1,ECM1,CCND2,ESR1,NFKBIE,TRF,CLN3,IKZF4,TAF13,L3MBTL3,CNN2,TCEANC,AHI1,PDPK1,DUSP16,SP4,PIK3CA,DEPDC1A,SUV420H2,MEX3D,RUNX2,ZFP280D,PIF1,PINK1,CENPK,ATF5,IGF2BP2,TRIP4,RHBDD1,ADNP2,GTF2B,ZFP748,PBRM1,HEXIM2,SPIB,TDRD7,CHAF1B,PBX2,NDST2,RBL1,NFE2,ZFYVE1,ZFP667,ZMYM2,MVP,NHLRC1,MXD1,ELAVL1,FLCN,ANKRD27,MDFIC,HMG20B,CREB3L1,CYTH3,EDN3,IMPACT,ITGB3BP,GTF2F1,NFIC,PPARGC1B,DUSP10,NR1D2,ZXDC,BCL2L11,CAMKK2,ZKSCAN14,TRIB3,IFT46,BBS4,CHMP5,GPD1L,CREB3,ZFP771,SERPINA3G,PRR13,ATXN7L3,TLR2,APLP2,GATAD2B,TAB2,PIK3IP1,PKNOX1,CTSB,LEMD3,ZFP592,DNAJB6,GGA3,EIF4EBP1,FBP1,ZFP202,MBD4,LIG3,CAST,HERPUD1,ARRB2,MLLT10,NEDD4L,TRIM37,GIT1,LPIN1,NLRC5,PPM1A,TAX1BP3,ZFP316,ICAM1,ATXN7,DGCR8,PRKD2,HMG20A,PHF20,HIP1,PRKAR2B,MED12L,COMMD7,CTBP2,TGFBR3,SP100,NUP62,MTF1,XIAP,IRF3,EGR3,NR4A2,SEMA4D,GBA,CPEB2,CSK,CHURC1,ZEB2,FNIP2,BRD8,CEBPB,GIPC1,ZFPM1,UBQLN4,THRA,NOSTRIN,MYCN,GADD45B,ZFP687,RAP1GDS1,ITGB1,HIVEP2,CREB3L2,AKAP13,BID,SIRT7,HACE1,CNPY2,INPP5K,ARAP3,STK3,ASXL1,MYC,WWP2,NFKBIZ,KAT5,ESRRA,RASAL3,ASCC3,IKBKG,KLF16,IRAK3,EZH1,FURIN,RGS12,CTDSP1,FHIT,TGFB1,TOLLIP,PPP1R14B,DPF3,ATAD1,TBC1D15,SIAH1A,NR1H2,LPCAT1,SQSTM1,MPV17,ZBTB44,MRE11A,INSIG2,MAVS,ZFP768,HBEGF,ZSCAN29,CHMP1A,SFI1,STRADA,LMO2
GO:0045087	Innate immune response	3.84e-08	191	33	GBP1,GBP2,CD74,TMEM173,IRF7,GBP3,SLC11A1,GCH1,OASL2,CLEC2D,DDX58,C3,ISG20,SIN3A,HERC6,ULBP1,IFIT1,NLRP10,STX11,PARP9,LGALS3,IRGM1,PDPK1,ATG12,ZBP1,PIK3AP1,DUSP10,GBP7,ARF6,TLR2,ARRB2,RAB27A,NLRC5
GO:0035556	Intracellular signal transduction	2.28e-06	1382	112	GSN,CD74,JUN,LATS2,RGS2,SLC11A1,TGM2,EPS8,MUC1,ABCA1,ASB11,RND2,DDX58,PJA2,C3,E2F2,DUSP1,DOCK5,CENPJ,WSB1,FGF23,RASIP1,ITSN1,DGKA,RAB7L1,HMOX1,GPX1,ERBB3,ZFP36,GADD45A,RAB34,UBR2,FGD6,ECM1,ESR1,TRF,PDPK1,DUSP16,PIK3CA,DEPDC1A,PINK1,PIK3AP1,ARL3,DOK3,RAB13,SOCS6,FLCN,RAP1GAP2,MDFIC,RAB37,NBR1,CYTH3,EDN3,RAB31,DUSP10,ARF6,BCL2L11,EPS8L2,TRIB3,ADA,SELP,GPD1L,NFKBIB,TLR2,NSMCE1,PIK3IP1,EIF4EBP1,IKBKE,FBP1,MBD4,ARRB2,RAB27A,GIT1,RGL1,SLC7A1,TMED4,PPM1A,TAX1BP3,ICAM1,PRKD2,UNC13B,TGFBR3,NUP62,AKR1B3,IRF3,SEMA4D,GBA,CSK,ZEB2,FNIP2,RAP1B,PGAP2,CUL3,RND1,GADD45B,RAP1GDS1,ITGB1,INPP5K,ARAP3,STK3,MYC,KAT5,RASAL3,IKBKG,IRAK3,FHIT,TGFB1,TBC1D15,SIAH1A,SQSTM1,PLCH1,MAVS
GO:0009966	Regulation of signal transduction	4.54e-05	1643	122	PF4,CD74,JUN,IRF7,LATS2,BMF,RGS2,TGM2,PRICKLE1,MUC1,ABCA1,SGK3,TRAF1,PJA2,C3,DUSP1,TRP53INP1,RAMP1,CENPJ,TNFRSF23,FGF23,RASIP1,ITSN1,HMOX1,GPX1,ERBB3,RBPMS,UBR2,FGD6,ECM1,ESR1,TRF,PDPK1,DUSP16,RUNX2,PINK1,ZBP1,GPSM1,PIK3AP1,MYO1C,MACF1,SOCS6,FLCN,RAP1GAP2,MDFIC,NBR1,CYTH3,EDN3,SH3GLB1,PPARGC1B,DUSP10,ARF6,BCL2L11,USP34,TRIB3,ADA,SELP,GPD1L,NFKBIB,TLR2,APLP2,PIK3IP1,LEMD3,FBP1,ARRB2,GIT1,RGL1,SLC7A1,TMED4,NLRC5,PPM1A,TAX1BP3,ICAM1,ATXN7,PRKD2,TGFBR3,NUP62,AKR1B3,XIAP,IRF3,SEMA4D,GBA,INTU,CSK,ZEB2,PGAP2,CUL3,GIPC1,THRA,GADD45B,RAP1GDS1,ITGB1,AKAP13,BID,TRIM68,INPP5K,ARAP3,STK3,ASXL1,MYC,RASAL3,IKBKG,IRAK3,FURIN,RGS12,TGFB1,DUT,TBC1D15,SIAH1A,SQSTM1,MAVS,HBEGF,MFNG,TLR4,TRIM30A,IFT80,TERF2IP,OTUD7B,ID1,PTPRJ,CSF2,CASP2
GO:0051702	Interaction with symbiont	7.01e-05	39	11	GBP1,GBP2,JUN,GBP3,SP1,NCF1,GPX1,ZFP639,GBP7,ICAM1,INPP5K
GO:0016043	Cellular component organization	1.00e-04	3338	214	PF4,GSN,CD74,JUN,TNFRSF9,LATS2,BMF,RGS2,SLC11A1,BTG2,TGM2,EPS8,TSSK6,AURKC,TRP53INP2,MUC1,ARID5A,EGR2,GCH1,MECP2,DPPA2,ABCA1,JHDM1D,SPINT1,SFMBT1,C3,LSP1,TRPV2,JDP2,ATP6V1D,MALAT1,RAMP1,TUBB2A,TRIM9,CENPJ,MMP9,CHRNB1,HMOX1,GPX1,DYNLT3,ZFP639,SIN3A,CDKN2C,FHOD3,CCNG2,PRMT7,ST7L,GADD45A,RAB34,UBR2,CEP97,SERAC1,NEU1,CLU,LGALS3,VPREB1,FBXO4,ESR1,TRF,CLN3,TLN1,4922501C03RIK,L3MBTL3,CNN2,AHI1,PDPK1,TPP1,OPTN,ATG12,SUV420H2,NDUFAF3,PIF1,PINK1,ADNP2,KLHL17,MAD2L1BP,PBRM1,CHAF1B,SEMA4A,ARL3,SGCB,MYO1C,MACF1,RBL1,WIPI1,9430023L20RIK,NFE2,TRPM7,FLCN,HMG20B,CGRRF1,COL5A1,TUBB1,NBR1,EDN3,ITGB3BP,RAB31,RPGR,FBLIM1,SH3GLB1,PPARGC1B,HIST2H2BE,TUBA8,ARF6,BCL2L11,FCHO2,LIPA,IFT46,BBS4,SELP,ADD2,TSGA10,CHMP5,MUM1,CREB3,BBS12,ATXN7L3,TLR2,IMMP1L,APLP2,NME7,DNAJB6,EIF4EBP1,FBP1,HIST3H2A,KCTD6,LIG3,DHRS4,ARRB2,NEDD4L,TRIM37,LPIN1,PPM1A,ICAM1,ATXN7,H2AFV,PRKD2,HMG20A,UNC13B,SMG6,PHF20,HIP1,TGFBR3,NCDN,CYFIP1,PODXL,NR4A2,SEMA4D,TSGA14,INTU,CSK,COL23A1,MARK4,BRD8,CUL3,NBEAL2,ZFPM1,THRA,QRSL1,NAIF1,RND1,RAP1GDS1,ITGB1,CFL2,BID,SIRT7,HACE1,INPP5K,ARAP3,2700049A03RIK,NEK3,ASXL1,MYC,KDM6A,KAT5,PCM1,PFKM,ATL3,IRAK3,CENPT,EZH1,MPP5,TK2,TUBB6,TGFB1,DUT,DPF3,ATAD1,NR1H2,SQSTM1,MPV17,MRE11A,MAVS,ABCA4,DHODH,HBEGF,CHMP1A,STRADA,PPP2R4,LPHN1,ALMS1,TUBD1,DCTPP1,TRIM30A,IFT80,TERF2IP,TPST2,DGAT1,MSL1,SRGN,PEX16,EPB4.1L2,CAND1,BET1
GO:0035458	Cellular response to interferon-beta	1.00e-04	24	6	GBP1,GBP2,TMEM173,GBP3,IFI203,IFIT1

GO:0034613	Cellular protein localization	2.54e-03	727	62	ARL6IP1,CD74,JUN,TMEM173,LATS2,GBP3,SLC11A1,PRICKLE1,EGR2,DDX58,GNPTG,ATP6V1D,RAMP1,NCF1,MORC3,SIN3A,RBPMS,KDELR3,SERAC1,STX11,TLN1,AHI1,PDPK1,OPTN,COPG2,MYO1C,PTTG1IP,MACF1,RAB13,FLCN,MDFIC,RAB31,SH3GLB1,ARF6,FCHO2,BBS4,GPD1L,CREB3,NFKBIB,TLR2,IMMP1L,NME7,SNX16,DNAJB6,GGA3,RAB27A,KPNB1,STXBP4,PPM1A,TAX1BP3,AP2A2,CSK,GIPC1,THRA,ITGB1,BID,INPP5K,PCM1,MPP5,TGFB1,MAVS,STRADA
GO:0006886	Intracellular protein transport	6.96e-03	454	41	ARL6IP1,CD74,JUN,TMEM173,GBP3,SLC11A1,PRICKLE1,EGR2,DDX58,GNPTG,RAMP1,NCF1,RBPMS,SERAC1,STX11,OPTN,COPG2,MYO1C,PTTG1IP,MACF1,RAB13,MDFIC,RAB31,CREB3,NFKBIB,TLR2,IMMP1L,SNX16,GGA3,RAB27A,KPNB1,STXBP4,PPM1A,AP2A2,CSK,GIPC1,THRA,ITGB1,BID,INPP5K,TGFB1
GO:0060340	Positive regulation of type I interferon-mediated signaling pathway	7.84e-03	8	5	IRF7,ZBP1,NLRC5,IRF3,MAVS
GO:0044699	Single-organism process	8.63e-03	8909	475	NEO1,PF4,GBP1,PIK3R3,GSN,ARL6IP1,MADCAM1,GBP2,SIGLEC5,SERPINF1,CD74,JUN,TMEM173,IRF7,FAM129A,TNFRSF9,LATS2,BMF,GBP3,RGS2,SLC11A1,CNR2,SLC39A8,BTG2,TGM2,PRICKLE1,DDC,EPS8,MYL4,IFI203,TSSK6,AURKC,USP2,TRP53INP2,TMC4,SPATA6,MUC1,ARID5A,EGR2,GCH1,MECP2,CIDEB,DPPA2,ANXA9,CCRL2,SGPL1,ABCA1,SLFN5,FOS,ASB11,SGK3,P2RY14,RAB11FIP2,SP1,JHDM1D,CLEC2D,SPINT1,RND2,PCYT1B,TRAF1,SCOC,SFMBT1,KLF6,DDX58,PJA2,C3,LSP1,E2F2,TRPV2,DUSP1,CTSS,LYZ1,GNPTG,SEPP1,JDP2,CELA1,TRP53INP1,ATP6V1D,MR1,MALAT1,2610002M06RIK,CNGA1,RAMP1,NCF1,PPP1R15A,DOCK5,TUBB2A,PPP3CB,CASP4,TRIM9,CENPJ,LPAR6,SLC14A1,WSB1,MMP9,TNFRSF23,FGF23,MORC3,CEBPD,CREM,RASIP1,CHRNB1,ITSN1,CSRNP2,CD79B,UGDH,DGKA,RAB7L1,HMOX1,GPX1,DYNLT3,ZFP639,SIN3A,ULBP1,CDKN2C,ABAT,FHOD3,IFIT1,SLCO2B1,PLIN2,NLRP10,ERBB3,0610031J06RIK,CCNG2,PRMT7,RBPMS,ZFP36,ST7L,NAPSA,GADD45A,KDELR3,RAB34,UBR2,RHPN2,ABCB1B,FGD6,CEP97,CBFA2T3,SERAC1,TNNI3,GP9,NEU1,CLU,STX11,TRPC2,PARP9,ARSB,LGALS3,BCL9,VPREB1,SLC16A4,FBXO4,FBXO6,TXNDC16,CCPG1,RNF167,ECM1,CCND2,IL2RB,EBI3,ESR1,NFKBIE,TRF,CLN3,MPG,RNF114,TLN1,2610002J02RIK,DNASE2A,4922501C03RIK,L3MBTL3,CNN2,AHI1,HEMGN,PDPK1,DUSP16,PHC1,TPP1,CTNS,OPTN,SP4,HSPB11,PIK3CA,DEPDC1A,ATG12,FFAR2,SUV420H2,RUNX2,NDUFAF3,PIF1,PINK1,ATF5,SLC18A1,ZBP1,TYROBP,RHBDD1,ADNP2,KLHL17,ACTA2,MAD2L1BP,PBRM1,GPSM1,HEXIM2,SPIB,PIK3AP1,SLC38A9,ATP6V1E1,TDRD7,B3GNT5,CHAF1B,SEMA4A,GMPR2,MND1,PBX2,ARL3,SGCB,NDST2,DOK3,IFI27L2A,COPG2,MYO1C,PTTG1IP,MACF1,ARHGAP39,RAB13,RBL1,WIPI1,MAP3K8,9430023L20RIK,NFE2,F2RL2,SOCS6,MVP,TRPM7,FLCN,RAP1GAP2,ANKRD27,PLEKHB1,MDFIC,HMG20B,RAB37,CREB3L1,CGRRF1,COL5A1,TUBB1,NBR1,CYTH3,EDN3,ITGB3BP,RAB31,OTOS,RPGR,FBLIM1,TDP1,NFIC,SLCO4A1,SH3GLB1,PPARGC1B,TSPAN8,NPEPPS,HIST2H2BE,TUBA8,SLC43A2,DUSP10,GBP7,NR1D2,ARF6,HVCN1,BCL2L11,EPS8L2,AVL9,FCHO2,USP34,TRIB3,LIPA,ADA,IFT46,BBS4,SELP,ADD2,TSGA10,CHMP5,SZT2,MUM1,GPD1L,CREB3,SERPINA3G,NFKBIB,BPGM,TNNT1,ATXN7L3,TLR2,PVR,IMMP1L,SLC12A5,APLP2,NSMCE1,TAB2,PIK3IP1,TRIP11,PKNOX1,CTSB,NME7,RDH12,LEMD3,SNX16,DNAJB6,TCN2,GGA3,CSPP1,EIF4EBP1,BC004004,ITGA2B,IKBKE,LGALS8,FBP1,HIST3H2A,TMEM18,KCTD6,PHC2,MBD4,LIG3,CAST,NUDT2,HERPUD1,ARRB2,MYLPF,NEDD4L,RAB27A,GIT1,LPIN1,RGL1,SLC7A1,KPNB1,STXBP4,TMED4,NLRC5,PPM1A,TAX1BP3,C8G,ICAM1,ZFYVE26,GBA2,ATXN7,H2AFV,DGCR8,USP42,PRKD2,HMG20A,YIPF6,UNC13B,SMG6,RNF138,PHF20,HIP1,PRKAR2B,COMMD7,NAIP2,MEMO1,CTBP2,VEGFB,TGFBR3,TNFAIP2,NUP62,AKR1B3,NCDN,SLC41A2,ICAM4,NAIP5,CYFIP1,PODXL,XIAP,IRF3,EGR3,NR4A2,SEMA4D,TSGA14,GBA,INTU,YIPF5,AP2A2,CSK,CHURC1,MARK4,ZEB2,FNIP2,BRD8,RAP1B,ERAP1,CML1,PGAP2,SELL,CEBPB,CUL3,NBEAL2,GIPC1,ZFPM1,THRA,DPCD,ATP6V0A1,QRSL1,OSBPL11,NAIF1,MYCN,RND1,GADD45B,LTB4R2,RAP1GDS1,ITGB1,HIVEP2,ABCB6,CREB3L2,AKAP13,SLC35A5,CFL2,BID,TRIM68,SIRT7,NIPSNAP1,HACE1,CNPY2,HRC,INPP5K,ARAP3,2700049A03RIK,NEK3,STK3,ASXL1,MYC,KDM6A,SPNS2,WWP2,CHIC2,KAT5,PCM1,ESRRA,RASAL3,LYZ2,S100A6,ASCC3,SLC48A1,PFKM,IKBKG,ATL3,KLF16,IRAK3,CENPT,EZH1,RNF19B,MPP5,TK2,TUBB6,FURIN,RGS12,CTDSP1,FHIT,GNPDA1,TGFB1,TOLLIP,DUT,A230046K03RIK,DPF3,LTB4R1,ATAD1,TBC1D15,SIAH1A,NR1H2,LPCAT1,SQSTM1,MPV17,EMILIN2,MRE11A,INSIG2,OSTM1,EXOC3L
GO:0032606	Type I interferon production	9.86e-03	38	10	TMEM173,IRF7,GBP3,DDX58,TLR2,IRF3,MAVS,TLR4,POLR3C,IFNAR1
GO:0010935	Regulation of macrophage cytokine production	4.33e-02	10	5	CD74,TLR2,IRAK3,TGFB1,TLR4
GO:0061082	Myeloid leukocyte cytokine production	4.46e-02	16	6	CD74,HMOX1,TLR2,IRAK3,TGFB1,TLR4
