# Supplementary figures and images for: Overexpression of the Transcription Factor Sp1 Activates the OAS-RNAse L-RIG-I Pathway
Source: PLoS One. 2015 Mar 4;10(3):e0118551. doi: 10.1371/journal.pone.0118551 (PMC4349862; doi:10.1371/journal.pone.0118551)

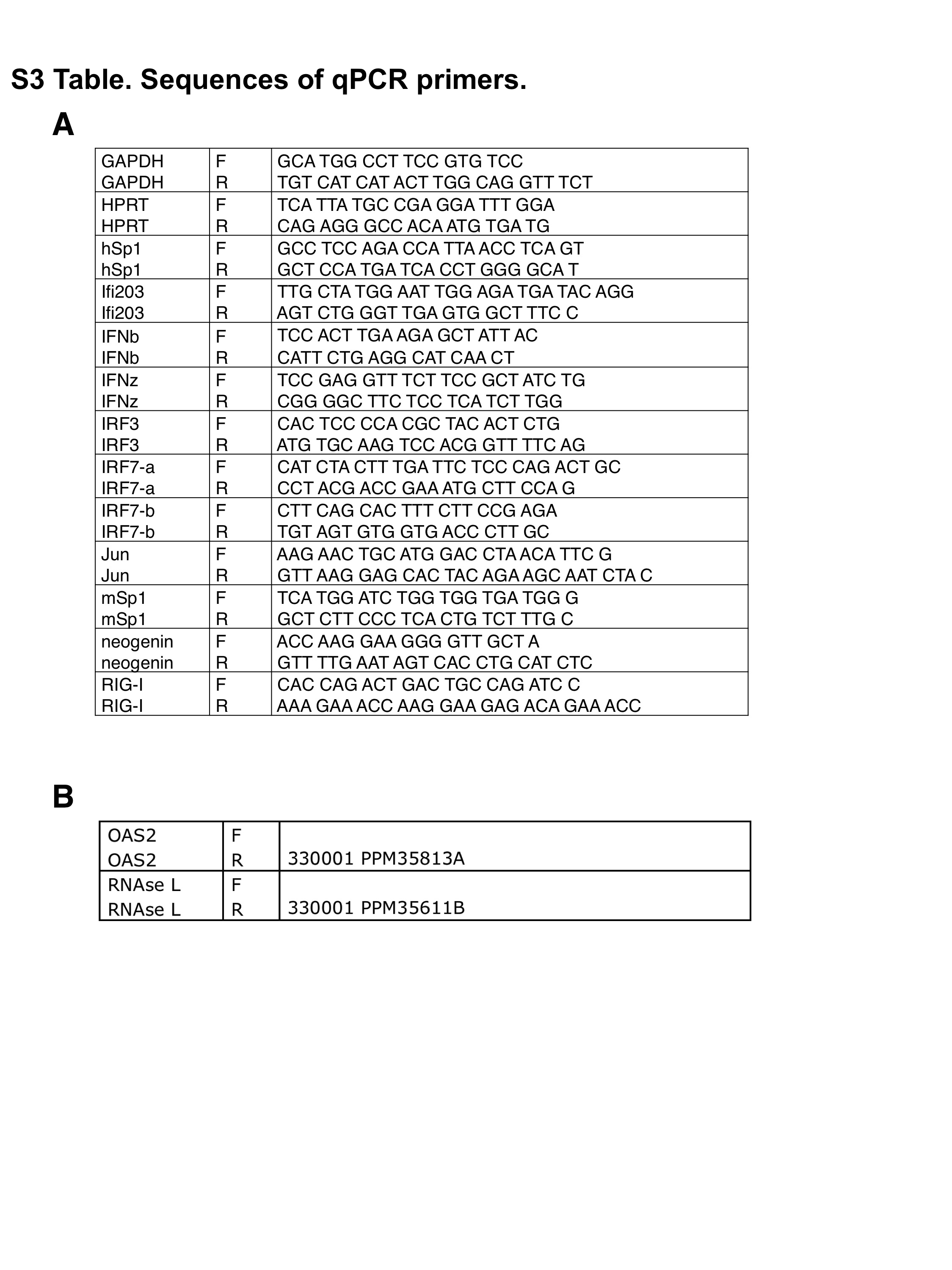

Supplement: S3 Table — (A) Primers used in Fig. 3, designed by our team. Primers for GAPDH, IFNβ and IRF7-b were used on StepOne+ Real-Time PCR Systems. Primers for HPRT, hSp1, RIG-I and IRF7-a were used on ABI PRISM 7000 Sequence Detection System. Ifi203, IFNz, IRF3, Jun and mSp1 were used on both systems. (B) Primers mix designed and validated by Qiagen, used in Fig. 5A and B. The catalog references are indicated. (TIFF) [file pone.0118551.s003.tiff]
